# Fat vs. Sugar: The Case for a Saturated Fat Tax in Italy

**DOI:** 10.1002/hec.4933

**Published:** 2025-01-12

**Authors:** Valeria di Cosmo, Silvia Tiezzi

**Affiliations:** ^1^ Department of Economics and Statistics “Cognetti de Martiis” University of Turin Turin Italy; ^2^ Department of Economics and Statistics University of Siena Siena Italy

**Keywords:** demand elasticities, exact affine stone index demand system, health benefits, unhealthy food taxes, welfare costs

## Abstract

When judging the distributional impact of unhealthy food taxes, what matters is not just how much low income people would pay but how much the such taxes would benefit or harm them overall. In this paper, we assess the consumer welfare impact of a fat tax net of its expected benefits computed as savings from weight loss. Using Italian data, we estimate a censored Exact Affine Stone Index (EASI) incomplete demand system for food groups, simulating changes in purchases, calorie intake, consumer welfare, and the monetary value of short‐run health benefits. While the Italian government has proposed a sugar tax, we show that there is no significant excess consumption of added sugars among Italian adults. Instead, excessive fat consumption is more prevalent, making a fat tax a more compelling and effective solution to address diet‐related health risks. Our results suggest costs from fat taxation are larger than benefits at all income levels. As a fraction of income, the net impact would be slightly regressively distributed.

## 1 Introduction

Sin taxes, or corrective taxes, are implemented in many countries on products like cigarettes, alcohol, soft drinks, and junk food to address their overconsumption (Cawley et al. 2019; Wright et al. 2017). These taxes aim to improve social welfare by reducing consumption and mitigating associated healthcare costs.

Taxing unhealthy foods is a contentious policy due to debates over its effectiveness and potential regressive impacts. Effectiveness concerns whether the tax can influence behavior, specifically reducing the consumption of unhealthy foods or nutrients to a socially optimal level in cases of market failure. A critical design question is whether to tax the product itself or the harmful nutrient responsible for health issues. For example, sugar taxes often target the sugar content of sugar‐sweetened beverages (SSBs), as health risks are directly tied to sugar consumption. This approach encourages firms to reformulate products and consumers to choose lower‐sugar alternatives. Notably, sugar taxes are frequently levied as a fixed amount per liter of beverage. For instance, Italy has debated and delayed the implementation of a volumetric sugar tax. However, this approach may fall short of maximizing health benefits since it offers limited incentives for consumers to switch to low‐sugar options or for producers to reformulate products. The regressive nature of sin taxes is another concern, as they disproportionately affect lower‐income individuals who tend to consume unhealthy goods at higher rates. However, if lower‐income groups reduce consumption the most in response to price increases, sin taxes might ultimately benefit disadvantaged groups more. In such cases, if welfare gains exceed the costs, the regressivity argument weakens. Evaluating the overall welfare and distributional effects of sin taxes requires careful consideration of both costs and benefits. Yet, the evidence on net welfare impacts remains limited (Allcott, Lockwood, and Taubinsky 2019b), with much of the literature focusing on either costs or benefits—rarely quantifying benefits in monetary terms.

This paper examines the net welfare effects of a nutrient tax on saturated fat for Italian households. We consider both the monetary health benefits of the tax, represented by savings from weight reduction, and the associated welfare costs. Our proposed tax targets foods high in saturated fats, as data reveal excessive consumption of these nutrients in Italy.

The dataset assembled for this study is unique in its breadth, combining multiple data sources. Household spending information on the entire consumption bundle is essential to accurately capture behavioral responses to price changes. While household scanner data is increasingly used for demand modeling (Dubois, Griffith, and O’Connell 2022), especially for specific product categories, our analysis extends to the entire array of consumption goods—including fresh foods and those not exclusively available through large‐scale retail channels. This broader scope allows us to analyze substitution and complementarity between food groups, a critical factor when assessing the impact of food taxes. However, such a comprehensive analysis is challenging with household scanner data. Instead, we utilize nationally representative pooled cross‐sections of Italian household consumption expenditures and associated price indices. This approach ensures inclusivity, covering purchases from all food retailers, including small retail stores and fresh produce transactions without barcodes. Combining this data with nutrient information from the European Institute of Oncology (IEO), we estimate a censored Exact Affine Stone Index (EASI) incomplete demand system (Lewbel and Pendakur 2009) for 16 food groups. This framework enables us to simulate changes in purchases, consumer surplus (measured using equivalent variation), and weight outcomes resulting from a tax on saturated fat. Additionally, we compute a 16×16 matrix of compensated price elasticities, which measure substitution (or complementarity) effects net of income changes—key to evaluating tax effectiveness.

Our counterfactual fat tax simulations focus on the “bad” nutrient most exceeding WHO guidelines in consumption, tailoring the tax to Italy's specific social and cultural norms. The analysis reveals that a sugar tax may not be the most effective policy for Italy, as there is little evidence of widespread consumption exceeding WHO recommendations for added sugar. Saturated fat emerges as a more viable target, and we estimate the tax rate required to reduce consumption to WHO‐recommended levels. To quantify short‐term tax benefits, we translate changes in consumption into bodyweight adjustments, following Hall et al. (2011). These weight changes are then monetized using data from the European Health Interview Survey (EHIS) and a two‐part model to estimate the impact of weight variations on individual healthcare expenditures. This provides a monetary metric for the tax's health benefits within a year of implementation.

Our contributions to the literature are threefold. First, unlike studies relying on household‐level purchase data,^1^ we use a sample of single‐household data^2^, to ensure a clear correspondence between expenditure on food categories and associated health benefits. Data from multi‐member households would obscure individual‐level health outcomes.

Second, we monetize the health benefits of weight loss by estimating the reduction in individual healthcare costs induced by the tax. In Italy, the National Health Service (Servizio Sanitario Nazionale, or SSN) provides universal health coverage, funded primarily through public expenditure.^3^ While primary and inpatient care are free, patients pay co‐payments for certain procedures. Our measure of monetary benefits accounts for reduced out‐of‐pocket health expenditures above SSN coverage. To accurately measure the marginal effect of weight changes on healthcare costs we follow Cawley and Meyerhoefer (2012) and use a two‐part model of medical expenditures (Jones 2000).

Third, we evaluate both costs and benefits of sin taxes in absolute terms and relative to income. Contrary to claims that sin taxes produce net benefits for lower‐income individuals, our findings show that a fat tax targeting a 30% reduction in saturated fat consumption imposes a small net welfare cost on the average Italian consumer. Distributionally, the tax is regressive: low‐income individuals experience proportionally larger net losses relative to their total expenditure. Interestingly, our results suggest that a modest increase in the value‐added tax (VAT) on certain food groups would yield welfare and distributional effects similar to those of a nutrient tax targeting saturated fat.

Our research contributes to several strands of literature on the effects of sin taxes. The first focuses on empirical studies employing demand system approaches. These studies use comprehensive frameworks to estimate price elasticities that capture consumers' behavioral responses to price increases, including reallocation of spending across the entire consumption basket. This allows for welfare changes to be expressed in monetary terms, incorporating these behavioral adjustments.

Works such as those by Chouinard et al. (2007); Zhen et al. (2014); Harkanen et al. (2014); Harding and Lovenheim (2017); Caro et al. (2020); McCullough et al. (2020), emphasize the role of substitution between food groups in assessing the impact of food and beverage taxes by estimating utility‐theoretic demand systems. However, these studies typically rely on household‐level data encompassing both adults and children, evaluating welfare changes at the household level or as per capita averages. While exceptions exist (e.g., Xiang, Zhan, and Bordignon (2018), which considers single‐household welfare costs. However, the demand system employed in this study is highly aggregated and does not allow for substitutions between food groups), they often aggregate demand at high levels, limiting analysis of substitutions between food groups. Moreover, health benefits—being inherently individual‐specific‐ are difficult to link directly to household purchases, leading most studies to focus solely on costs or benefits, often without expressing benefits in monetary terms. Our approach departs from these studies by using single‐household data, enabling precise alignment between purchases, consumption, welfare costs, and health benefits at the individual level. We also estimate health benefits in monetary terms, addressing a key gap in the literature.

A second related literature examines the monetary valuation of health benefits from unhealthy food taxes. Recent studies in high‐income countries like Australia, Canada, and the U.S. have found that lower‐income groups often experience equal or greater health benefits from these policies, measured as savings in healthcare expenditures tied to reduced disease incidence (Kao et al. 2020; Lal et al. 2017; Wilde et al. 2019). These studies typically focus on long‐term benefits, assuming zero substitution between taxed goods and other food groups, and often import elasticity estimates from external sources. As a consequence, there could be some misalignment between consumer costs and benefits. We diverge from this literature in two significant ways. First, we estimate both own‐ and cross‐price elasticities for our individual‐level sample, accounting for complementarities and substitutions across all food groups in response to taxation. Pure substitution effects are captured through compensated cross‐price elasticities, net of income effects. Second, we estimate short‐term tax benefits for each individual by calculating expected savings in out‐of‐pocket healthcare costs resulting from predicted weight changes 1 year after tax implementation.

Finally, our study engages with a smaller body of work that directly links consumer costs and benefits to evaluate the overall impact of sin taxes (Allcott, Lockwood, and Taubinsky 2019a; Dubois, Griffith, and O’Connell 2020). For instance, Allcott, Lockwood, and Taubinsky (2019a) provide a framework incorporating consumer bias correction, externalities, and revenue recycling in evaluating sugar taxes in the U.S., finding slightly regressive net gains. Similarly, Dubois, Griffith, and O’Connell (2020) use these estimates to show that a sugar tax in the U.K. would be only mildly regressive under lump‐sum redistribution. In contrast, our findings suggest that short‐term costs from a fat tax outweigh short‐term benefits at all income levels. Furthermore, as a fraction of income, the net impact is slightly regressive.

The remainder of the paper is organized as follows: Section 2 describes the data sources. Section 3 outlines the demand model, estimation procedure, and derived elasticities. Section 4 examines the welfare costs and distributional effects of our fat tax simulations. Section 5 discusses the monetary value of short‐term health benefits and evaluates the net welfare and distributional impact of the proposed fat tax. Section 6 concludes.

## 2 Data

### 2.1 Expenditures and Prices

We aim to include food expenditures from all food retailers, including small local shops, which contribute significantly to food spending in Italy. Italians purchase fresh produce not only from supermarkets but also from markets, bakeries, butchers, and other establishments (Cozzi 2008). To ensure comprehensive coverage, we use 5 cross‐sectional micro‐data sets from the Household Budget Survey (HBS) conducted by ISTAT from January 2014 to December 2018.^4^ The HBS data covers all food expenditures, regardless of retailer type, aligning with the aggregation level of our nutrient dataset. Each annual cross‐section contains monthly data from approximately 23,000 Italian households across 480 municipalities.^5^ The survey also includes detailed household and sociodemographic information, such as regional location, household size, and the gender, age, education, and employment status of each member.^6^ The HBS provides household‐level expenditure data. To link individual welfare costs and health benefits to expenditures, we focus on households with one member. However, to compare, we also estimate demand models and compute elasticities and welfare costs for households with two adults and a child aged 14 or younger.

Our final sample includes 12,369 individuals in single‐adult households, categorized by 21 regions and three urban types (metropolitan areas, medium cities, small cities). The sample of two adults and one child includes 4772 households. The HBS food consumption module records expenditure on about 200 items based on a seven‐day recall, which we aggregate into 16 food‐at‐home groups and one food‐away‐from‐home category, totaling 17 groups. These groups reflect typical Italian meals and food characteristics: alcoholic drinks; bread and pasta; cereals and rice; eggs and milk; fat and cheese; fish; food away from home; fruit; oil; non‐sweetened/non‐alcoholic drinks; processed meat; poultry; red meat; sugar‐sweetened beverages; snacks and sweets; vegetables; and others. The “other” category is used as a numeraire in our demand system (LaFrance and Hanemann 1989; Hanemann and Morey 1992) and to calculate total consumption expenditure and budget shares. Descriptive statistics for the average budget share and log prices are provided in Supporting Information S1: Table B1 in Appendix B.^7^

Since the HBS does not include price data, we use monthly consumer price indices (set to 100 in 2015) from ISTAT, spanning January 2014 to December 2018. These indices are part of the Harmonized Consumer Price Index (HCPI) compiled by Eurostat. We associate each expenditure category in the HBS with its corresponding price index, following the COICOP classification developed by the United Nations Statistics Division. For aggregation, we use a 5‐digit COICOP breakdown, which provides a granular price disaggregation matching our HBS expenditure categories. To address the issue of price collinearity in disaggregated demand systems, we compute Stone‐Lewbel prices (Lewbel 1989) for the food groups in our demand system. By assuming constant expenditure shares within each group, we weight the prices of individual goods within each group by their expenditure shares, introducing cross‐sectional variation into the price indices.^8^

### 2.2 Nutrients

The nutrient content (calories, fats, and sugars) of our food groups was calculated using conversion factors from the 2015 Food Composition Database for Epidemiological Studies in Italy (FCDES), provided by the European Institute of Oncology (EIO).^9^ This database enables nutrient values to be determined per kilogram of each food group^10^. The EIO database provides detailed nutrients data at the product level, while the HBS dataset categorizes expenditures more broadly. To align the two, we aggregated EIO data to match 5‐digit COICOP categories, assigning nutrient values to our food groups based on these aggregates. While this broad categorization does not allow to look at within‐group substitutions, potentially yielding conservative welfare costs estimates, the HBS dataset was chosen for its comprehensive coverage of individual food expenditures, making it the most suitable for our analysis.

Table 1 presents the sugar, saturated fat, and calorie content per kilogram of the final product. As expected, sugar levels are particularly high in sweets, snacks, and sweetened beverages, while saturated fat is most prevalent in fats, cheeses, oils, and processed meats.

**TABLE 1:** Nutrients (grams) and Kcal per kg of food group.

Food groups	Obs	Sugar	Saturated fats	Kcal
Vegetables	10,638	0.0	1.4	647
Fruit	10,638	30.60	18.0	1762
Pasta and bread	10,638	0.0	5.2	2899
Cereals and rice	10,638	0.0	4.8	2938
Eggs and milk	10,638	12.5	34.9	1710
Fish	10,638	3.3	11.3	1228
Poultry	10,638	0.1	25.4	1675
Red meat	10,638	0.3	29.5	1515
Processed meat	10,638	2.5	71.4	2788
Fat and cheese	10,638	0.0	160.5	3453
Oil	10,638	1.4	216.6	8660
Sweets and snacks	10,638	113.7	30.6	3056
Sweetened beverages	10,638	37.6	0.1	414
Other drinks	10,638	2.8	9.8	860

*Note:* Sugar, saturated fats and Kcalories contents per kg of each food category calculated from the 2015 release of the European Institute of Oncology FDCES. The nutrients' contents of food‐away‐from‐home could not be assessed due absent information on the food items in each purchased meal.

We use the WHO's recommended reference value of 30 g/day for sugar and saturated fat intake (WHO (2018); WHO (2015)) to calculate overconsumption. This is defined as the difference between the average daily intake of sugar (excluding fruit‐derived sugar) and saturated fats in our sample and the reference value (Griffith et al. (2016)).

Our data shows only a slight excess sugar consumption among single adults. Interestingly, a similar trend is observed in households with two adults and one child, despite approximately 20% of Italian children being overweight before the COVID‐19 crisis (WHO 2022). Recent research (Spinelli et al. 2023) indicates that the percentage of Italian children consuming unhealthy foods like sugary drinks more than 3 days a week is below the global average. This aligns with findings that children's dietary habits are shaped by complex, interacting factors, including shared environments, norms, and behaviors (Crudu, Neri, and Tiezzi 2021). These results question the effectiveness of food taxes in addressing child obesity in Italy, suggesting a need for more comprehensive interventions.^11^

**FIGURE 1:**
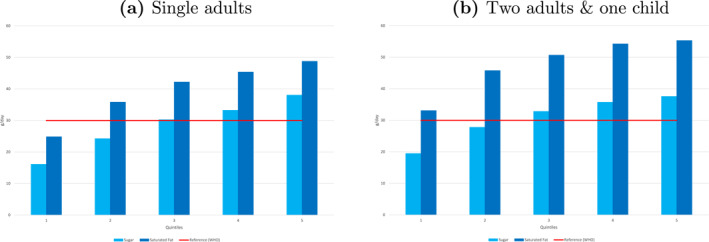
Sugar and saturated fats overconsumption by income quintile. Average daily intake (grams/day) of sugar and saturated fats for single adults (a) and for equivalent adult (b) across quintiles of total expenditure. Horizontal lines represent the threshold recommended by the WHO (30 g/day).

Figure 1 illustrates no overconsumption of sugar among individuals below the fourth income quintile (Figure 1a) or the third quintile for adults in households with two adults and one child (Figure 1b). The sample's average sugar intake (28 g/day) falls below the WHO‐recommended threshold (30 g/day) for single adults (Figure 1a) and is only slightly above the threshold (30.67 g/day) for households with two adults and one child (Figure 1b). This suggests that the forthcoming volumetric sugar tax in Italy may generate revenue but is unlikely to significantly reduce sugar consumption or improve public health.

In contrast, saturated fat intake averages 42 g/day for single adults (Figure 1a) and 47.8 g/day per adult equivalent in households with two adults and one child (Figure 1b), exceeding the WHO threshold (30 g/day) by approximately 33%. Excess fat consumption increases with income, with higher quintiles showing greater excesses. This pattern reverses the usual trend observed in other contexts, where lower‐income groups consume more unhealthy nutrients. Similar patterns are seen in other household types (see Supporting Information S1: Figure 1 in Appendix A), where higher‐income families consume more unhealthy nutrients, though intra‐household consumption allocation cannot be assessed.

### 2.3 Health Expenditures and Health Related Variables

Data on individual body weight and health‐related variables in our sample are from the 2015 Italian module of the European Health Interview Survey (EHIS), conducted every four years across EU member states. To align health expenditure from HBS data with individual body weight and health variables, we employ the matching method of Rubin (1986) and Moriarity and Scheuren (2003), detailed in Supporting Information S1: Appendix E.

## 3 The Demand Model

We estimate an incomplete Exact Affine Stone Index (EASI) implicit Marshallian demand system (Lewbel and Pendakur 2009) including 16 food groups and a composite numéraire that incorporates all other consumption goods and services plus a residual food category.^12^ The demand system's estimated parameters can be used to provide exact measures of changes in welfare, unlike those of conditional demand models (LaFrance and Hanemann 1989; Hanemann and Morey 1992). Conditional demand systems underestimate the degree of substitution among expenditure groups after a price change (Zhen et al. 2014), because weak separability between food expenditure and that of all other consumption implies that only substitutions among food groups are taken into account. An incomplete demand system, on the other hand, produces unconditional predictions of demand responses to a simulated price change.

One potential problem in estimating a demand system with household level data is the existence of zero observations due to infrequent purchase of highly disaggregated food categories. We adopt Shonkwiler and Yen (1999) two‐step estimation procedure to address this issue. After modifying the EASI incomplete demand system to account for censoring, the implicit Marshallian budget shares equations to be estimated are:

(1) $$w^{j}=\Phi \left(v^{\prime }\lambda^{j}\right)\left[\sum_{r=1}^{R}b_{r}^{j}{\left(y\right)}^{r}+\sum_{t=1}^{T}g_{t}^{j}z_{t}+\sum_{k=1}^{J}a^{jk}lnp^{k}\right]+\tau^{j}\phi \left(v^{\prime }\lambda^{j}\right)+\varepsilon^{j}$$

(2) $${\left(y\right)}^{r}=\left(\ln x-\sum_{j=1}^{J}w^{j}\ln p^{j}+\frac{1}{2}\sum_{j=1}^{J}\sum_{k=1}^{J}a^{jk}\ln p^{j}\ln p^{k}\right)$$

where $w^{j}$ is the budget share of commodity j; J is the number of goods with the $J^{th}$ good being the composite numéraire; y is real household income; R is the highest order of the polynomial in y to be determined empirically; $p^{k}$ is the price index of the $k^{th}$ good; *T* is the number of exogenous demand shifters; $z_{t}$ is the $t^{th}$ demand shifter; $b_{r}^{j}$, $g_{t}^{j}$ and $a^{jk}$ are parameters to be estimated; and $\varepsilon^{j}$ is the error term. Denoting the vector of predictors of positive consumption and the vector of their associated parameters by v and λ for equation j, $\Phi \left(v^{\prime }\lambda^{j}\right)$ and $\phi \left(v^{\prime }\lambda^{j}\right)$ are the normal cumulative distribution and probability density functions, respectively, related to the first‐stage probit equations introduced to correct the bias in the coefficients of the EASI model caused by censoring. Finally, x in Equation (2) is nominal total consumption expenditure.

To ensure integrability of the demand equations we impose the theoretical restrictions of homogeneity: $\sum_{k=1}^{K}a^{jk}=0$ for all j=1,…,J; symmetry: $a^{jk}=a^{kj}$; and adding up. Adding up requires that the sum of the *J* coefficients associated with the constant of each share equation (denoted $z_{0}$) is equal to one: $\sum_{j=1}^{J}g_{0}^{1}=1$; and that the sum of the *J* coefficients associated with any other variable in the budget shares equations is equal to zero: $\sum_{j=1}^{J}a^{jk}=0,\;k=1,\text{…},J$;$\sum_{j=1}^{J}b_{r}^{j}=0,\;r=1,\text{…},R$; $\sum_{j=1}^{J}g_{t}^{j}=0,\;t=1,\text{…},T$.

The EASI demand system is nonlinear and endogenous. Nonlinearity arises from the fact that $b_{r}$ multiplies a power of y. Endogeneity is due to the budget‐shares $w^{j},j=1,\text{…},J$ being on both sides of the system of equations. Estimation is further complicated by the presence of censoring. However, like the QAID, the EASI demand system can be approximated using linear‐in‐the‐parameters equations. The approximated model replaces y with $\overset{∼}{y}=\ln x-\sum_{j=1}^{J}w^{j}\ln p^{j}$, where $\overset{∼}{y}$ is the log nominal expenditures deflated by the Stone price‐index.^13^ To correct for endogeneity due to the introduction of budget shares into log real total expenditure we create an instrument for y constructed as logx deflated by a modified Stone price index where ${\bar{w}}^{j}$, the sample‐average budget share for food group j, replaces $w^{j}$ (Lewbel and Pendakur 2009): $\hat{y}=\ln x-\sum_{j=1}^{J}{\bar{w}}^{j}\ln p^{j}$. In addition to a constant, we specify a vector of demand shifters $z_{k}$.^14^ Descriptive statistics for these demand shifters are shown in Supporting Information S1: Table B2 in Appendix B.^15^ Engel curves are shown in Supporting Information S1: Appendix C.

### 3.1 Elasticities

**FIGURE 2:**
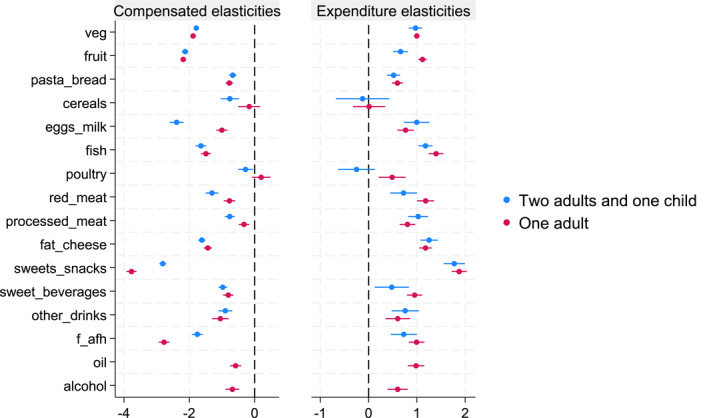
Own‐price compensated and expenditure elasticities. Compensated own‐price elasticities (left) for each food group for single households (red) and households with two adults and one child (blue). Expenditure elasticities (right) for each food group for single households (red) and households with two adults and one child (blue). For the latter, elasticities for oil and alcohol are not reported as the Lewbel procedure did not converge due to the small number of observations. Bootstrapped standard errors with 200 replications.

Behavioral reactions to price changes are measured using own‐ and cross‐price elasticities, which capture changes in the purchased quantities of food groups and their substitutions or complementarities.^16^ Our structural model allows a 16×16 matrix of 256 estimated price elasticities. Figure 2 highlights the main diagonal of the compensated elasticity matrix (left) and expenditure elasticities (right) for single adults (red dots) and households with two adults and one child (blue dots). All own‐price elasticities have the expected negative sign, with most statistically significant at 1% in both samples. The largest differences between household types are seen for eggs and milk, sweets and snacks, and food away from home. Families with children are more sensitive to price changes in eggs and milk but less responsive to changes in prices for sweets, snacks, and food away from home compared to single adults.

Among food groups, sweets and snacks exhibit the largest own‐price elasticity (−3.770 for single adults), meaning a 1% price increase would reduce quantity purchased by approximately 3.8%. Fat and cheese also show a large price elasticity (−1.432 for single adults), while sweetened beverages have an elasticity of −0.805, within the range reported in the literature (Finkelstein et al. 2010).^17^ Most expenditure elasticities are positive and significant at 1%, with most food groups classified as necessities (elasticity < 1). Fish, fat and cheese, and sweets and snacks are luxuries (elasticity > 1) in both samples.

We next focus on substitution and complementarities among food groups for single adults. Table 2 presents the compensated price and expenditure elasticities at the sample mean.^18^ Compensated elasticities capture substitutions net of income effects. Positive, significant cross‐price elasticities indicate substitutions, while negative ones indicate complementarities. Notably, increasing the price of sweets and snacks leads to substitutions with vegetables (0.202), alcohol (0.199), and food away from home (0.181), while showing complementarities with eggs and milk (−0.139), cereals (−0.411), sweetened beverages (−0.462), and other drinks (−0.182). Raising the price of sweetened beverages results in substitutions with fruit, fish, and food away from home (0.096, 0.019, 0.023) and complementarities with oil, sweets and snacks, and other drinks (−0.160, −0.112, −0.160). Increasing fat and cheese prices causes substitution with bread and pasta (0.091) and complementarity with fruit (−0.064).

**TABLE 2:** Compensated price elasticities and expenditure elasticities (single adults) ‐ sample means.

	Vegetables	Fruit	Pasta and bread	Cereals and rice	Eggs and milk	Fish	Poultry	Red meat	Processed meat	Fat and cheese	Oil	Sweets and snacks	Sweetened beverages	Other drinks	Alcohol	Food‐away‐from‐home
Vegetables	−1.883***	−0.002	0.133***	0.000	0.092***	−0.131***	0.005	0.087***	0.055**	0.007	−0.030*	0.202***	−0.016*	0.020	−0.070***	0.117***
Fruit	−0.003	−2.186***	0.032	−0.104***	−0.002	−0.028	0.010	0.060	0.120***	−0.064*	−0.016	0.044	0.096***	0.033	0.157***	0.348***
Pasta and bread	0.228***	0.037	−0.773***	−0.034	−0.004	−0.069	−0.098*	0.011	−0.239***	0.091**	−0.004	−0.045	0.007	−0.013	−0.044	0.162***
Cereals and rice	−0.002	−0.558***	−0.155	**−0.165**	0.114	−0.076	−0.087	−0.256	0.207	−0.017	−0.190*	−0.411**	−0.066	−0.009	0.388*	0.396**
Eggs and milk	0.208***	−0.003	−0.005	0.033	−1.003***	−0.131**	0.054	−0.226***	−0.290***	0.066	−0.037	−0.139**	0.006	0.022	0.039	0.185**
Fish	−0.236***	−0.034	−0.072	−0.017	−0.104**	−1.491***	−0.006	−0.036	0.066	0.020	−0.042	0.074	0.049*	−0.045	−0.072	0.161*
Poultry	0.013	0.019	−0.164*	−0.031	0.068	−0.009	**0.202**	−0.609***	−0.086	−0.139	−0.245***	0.161*	−0.050	0.027	−0.101	−0.060
Red meat	0.147***	0.068	0.011	−0.055	−0.168***	−0.034	−0.359***	−0.770***	−0.162**	−0.068	0.052	−0.008	−0.018	−0.103*	−0.162**	−0.029
Processed meat	0.101**	0.149***	−0.257***	0.048	−0.235***	0.068	−0.055	−0.177**	−0.324***	−0.098	−0.054	−0.062	−0.011	−0.233***	−0.090	0.045
Fat and cheese	0.013	−0.079*	0.098**	−0.004	0.053	0.021	−0.089	−0.074	−0.098	−1.432***	0.041	−0.044	−0.011	−0.069	−0.036	0.134*
Oil	−0.152*	−0.056	−0.010	−0.121*	−0.081	−0.118	−0.430***	0.154	−0.149	0.113	−0.582***	−0.056	−0.160***	0.164*	−0.050	0.208*
Sweets and snacks	0.275***	0.041	−0.036	−0.071**	−0.083**	0.056	0.077*	−0.006	−0.046	−0.033	−0.015	−3.770***	−0.112***	−0.107**	0.122**	0.555***
Sweetened beverages	−0.090*	0.364***	0.024	−0.047	0.016	0.153*	−0.099	−0.061	−0.034	−0.034	−0.179***	−0.462***	−0.805***	−0.388***	0.045	0.293***
Other drinks	0.046	0.052	−0.017	−0.003	0.023	−0.057	0.022	−0.141*	−0.293***	−0.086	0.076*	−0.182**	−0.160***	−1.048***	−0.053	0.192*
Alcohol	−0.154***	0.235***	−0.057	0.109*	0.038	−0.088	−0.079	−0.213**	−0.108	−0.044	−0.022	0.199**	0.018	−0.050	−0.680***	−0.377***
Food‐away‐from‐home	0.052***	0.104***	0.042***	0.022**	0.036**	0.040*	−0.009	−0.008	0.011	0.032*	0.018*	0.181***	0.023***	0.037*	−0.076***	−2.772***
**Expenditure**	0.998***	1.117***	0.597***	**0.010**	0.767***	1.398***	0.488***	1.182***	0.805***	1.180***	0.982***	1.877***	0.953***	0.604***	0.602***	0.994***

*Note:* The cells of each row show the price elasticity of the food group of the row due to a change in price of the food group of the column. For example, the third entry in the first column (0.228) is the percentage change in the demand for pasta and bread following a 1% increase in the price of vegetables. Expenditure elasticities of demand on the last row. * = *p* < 0.1; ** = *p* < 0.05; *** = *p* < 0.01. Bootstrapped standard errors with 200 replications in parentheses. Own‐price elasticities are bolded in the table.

Concerns about substituting high‐fat and high‐sugar foods (fat and cheese, sweets and snacks, sweetened beverages) with food away from home, whose nutritional content is unknown, are addressed by Table 2. Substitutions with food away from home are small (0.181 for sweets and snacks, 0.023 for sweetened beverages, and 0.032 for fat and cheese). Increasing the price of red meat reduces purchases of eggs and milk, poultry, processed meat, and alcohol (−0.226, −0.609, −0.177, −0.213, respectively). Similarly, price increases for processed meat cause substitutions and a complementarity with other drinks (−0.293), suggesting that higher red meat and processed meat prices may improve overall diet quality. Supporting Information S1: Table D2 in Appendix D shows compensated own‐price and expenditure elasticities for single adults at low and high total expenditure levels, serving as a proxy for income. As expected, low‐income individuals show stronger reactions to price changes across most food groups. However, higher‐income individuals, with greater consumption of fat and cheese, exhibit higher own‐price elasticity for these items (−1.633 vs. −1.088 for low‐income individuals). This challenges the narrative that lower‐income individuals consume more unhealthy nutrients and are more responsive to price increases.

## 4 Counterfactuals

In the main counterfactual experiment we use our demand estimates for single adults to simulate the introduction of a specific (s) tax (τ) proportional to the saturated fat content of each food group. Let $\eta^{j}$ denote the saturated fat content of 1 kg of food group *j*. We assume that the post‐tax price of commodity *j*, $p_{1,s}^{j}$, is related to pre‐tax price, $p_{0}^{j}$, according to:

(3) $$p_{1,s}^{j}=p_{0}^{j}+\tau \eta^{j}$$

**FIGURE 3:**
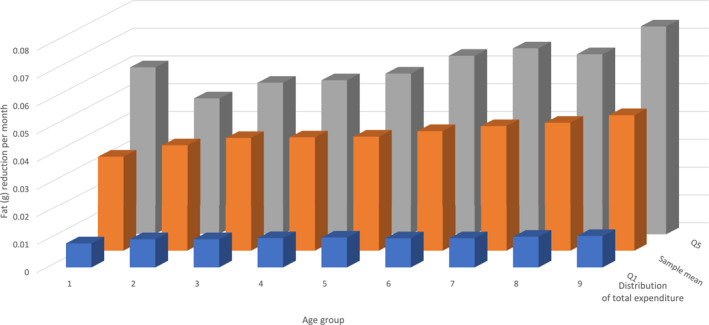
Reduction in saturated fat consumption (grams). Vertical axis: variation (in grams) of saturated fat consumption per month after the introduction of the fat tax; horizontal axis: distribution of age (age groups: 1 = 18–24, 2 = 25–29, 3 = 30–34, 4 = 35–39, 5 = 40–44, 6 = 45–49, 7 = 50–54, 8 = 55–59 and 9 = 60–64 years); third axis: distribution of total expenditure.

We select the rate of tax that results in a 30% decrease in saturated fat purchased assuming a 100% pass‐through of taxes to prices.^19^ For each food group j,j=1,…,J, the specific tax on saturated fat is:

(4) $$\tau \eta^{j}=\frac{-0.30}{\epsilon^{j}}p_{0}^{j}\eta^{j}$$

where $\epsilon^{j}$ is the own‐price compensated elasticity of quantity for group j ^20^. Table 3 shows the vector of percentage price variations after the introduction of the specific fat tax. The effectiveness of a fat tax can be evaluated by how much consumers decrease their fat consumption after the tax. Figure 3 shows the variation in saturated fat consumption (grams) per month after the tax across the distributions of age and of total monthly expenditure (our proxy for income). Across the age distribution, young consumers are equally likely to be affected by the policy as adults. Interestingly, young adults in the top quintile of the expenditure distribution achieve larger reductions in fat consumption compared to older age groups. However, young adults in the first quintile or at the sample mean of the expenditure distribution experience smaller fat reductions compared to older age groups. The fat tax achieves relatively large reductions in fat consumption among individuals with average (orange columns) and high (gray columns) total expenditure but is not successful at targeting individuals in the lowest quintile (blue columns) of the expenditure distribution.^21^

**TABLE 3:** Percentage price variations after the tax.

Food groups	Price variation (%)
Vegetables	0.021
Fruit	0.245
Pasta and bread	0.201
Cereals and rice	0.000
Eggs and milk	1.036
Fish	0.224
Poultry	0.000
Red meat	1.124
Processed meat	6.409
Fat and cheese	3.328
Oil	11.070
Sweets and snacks	0.241
Sweetened beverages	0.003
Other drinks	0.278
Alcohol	0.000
Food‐away‐from‐home	0.000

### 4.1 Consumer‐Welfare Costs and Redistribution

We use our demand estimates to compute the compensating variation (CV), a money metric measure of welfare change after a price change, defined as the minimum sum of money necessary to fully compensate a consumer after the price change. If $w_{0}$ is the baseline welfare level before any price change, CV is the sum of money necessary to make an individual indifferent to the change in tax policy: $CV=c\left(w_{0},p_{1}\right)-c\left(w_{0},p_{0}\right)$ where $c\left(w_{0},p_{0}\right)$ is the minimum cost of achieving $w_{0}$ at prices $p_{0}$, and $c\left(w_{0},p_{1}\right)$ is the minimum cost of attaining utility $w_{0}$ at the price vector $p_{1}$. To calculate the CV, we use the True Cost of Living (TCOL) index (Deaton and Muellbauer 1980), the ratio of the cost of achieving a given level of economic welfare after a price change to the cost of achieving the same level of economic welfare before the price change: $TCOL=\frac{c\left(w_{0},p_{1}\right)}{c\left(w_{0},p_{0}\right)}$. The CV and the TCOL are clearly related to each other: $CV=c\left(w_{0},p_{0}\right)\times \left(TCOL-1\right)$.

**FIGURE 4:**
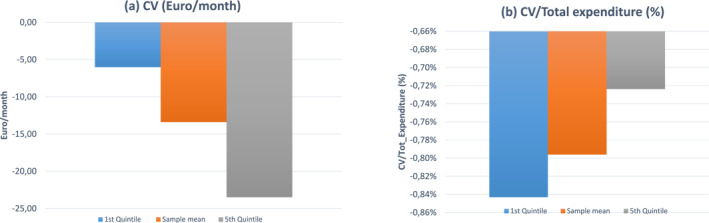
Compensating Variation (CV). (a) shows welfare costs in €/month across the distribution of total expenditure, our proxy for disposable income. Welfare costs are measured by the compensating variation (€/month) after a price change. (b) shows the distribution of welfare costs as a fraction of total expenditure.

The EASI log change in the TCOL index (Lewbel and Pendakur 2009) is calculated as:

(5) $$\ln\left(\frac{x_{1}}{x_{0}}\right)={\left(p_{1}-p_{0}\right)}^{\prime }w_{0}+0.5{\left(p_{1}-p_{0}\right)}^{\prime }\Gamma \left(p_{1}-p_{0}\right)$$

where $x_{1}$ is the post‐tax income necessary to maintain utility at the pre‐tax level; $p_{1}$ is the J×1 vector of new log prices after the tax is imposed, and Γ is a J×J matrix of parameters whose element $\Gamma_{ij}$ equals $a^{jk}$ in Equation (1). Equation (5) captures two effects of the fat tax on welfare. The first term on the right‐hand‐side is the Stone price effect that ignores any changes in budget shares of the taxed goods. The second term measures the effect on budget shares as a consequence of substitution. The total effect will be smaller than the Stone price effect if budget shares of the taxed goods decrease in response to the tax. Figure 4 illustrates the consumer‐welfare effects of the specific fat tax for single adults. The welfare loss increases with income. At mean income, CV is 13.40 €per month. Relative to income, proxied by total monthly consumption expenditure, the welfare loss is regressively distributed.^22^

## 5 Monetary Value of Weight Loss

Excess saturated fat consumption can lead to weight gain. We estimate the short‐run tax benefits by approximating the health benefits of weight loss (Hall et al. 2011; Lin et al. 2011; Harkanen et al. 2014; Xiang, Zhan, and Bordignon 2018). To do this, we calculate the fat tax's impact on food consumption by multiplying the matrix of uncompensated price elasticities (Supporting Information S1: Table D1 in Appendix D) by a vector of percentage changes in consumer prices. Table 4 (left) shows the relative demand changes, computed as $\frac{\left(q_{1}^{j}-q_{0}^{j}\right)}{q_{0}^{j}}=\epsilon_{j}\times \frac{\left(p_{1}^{j}-p_{0}^{j}\right)}{p_{0}^{j}}$, for each food group j. We then calculate the change in harmful nutrient intake following the tax, shown in Figure 3 by age and across the income distribution (using total expenditure as a proxy for disposable income). As noted, the tax significantly reduces fat consumption among those with average and high incomes but is less effective for individuals in the lowest expenditure quintile. Consequently, we expect health benefits to be more pronounced at higher income levels. To calculate individual weight changes due to reduced fat consumption, we use the rule of thumb from Hall et al. (2011), which suggests that a 100 kJ/day change in energy intake results in a 1 kg weight change, with half the change occurring within a year and 95% within 3 years. This estimate is based on dynamic simulation models predicting weight changes from energy balance interventions. Table 4 (right) shows the average weight reduction (1.72 kg) and the change in energy intake (kJ/day) 1 year after the fat tax. We also estimate the probability of transitioning from obese to overweight or overweight to normal weight after a 1.72 kg weight loss. Using the EHIS dataset, we find that 2.09% of obese individuals transition to overweight, and 5.94% of overweight individuals transition to normal weight.^23^

**TABLE 4:** Monthly percentage changes in quantities purchased (left); changes in body weight (kg) and daily energy intake (kJ) 1 year after the fat tax (right).

Food groups	Variation (%)
Vegetables	−0.037
Fruit	−0.441
Pasta and bread	−0.137
Eggs and milk	−1.714
Fish	−0.439
Red meat	−1.776
Processed meat	−7.313
Fat and cheese	−5.251
Oil	−15.222
Sweets and snacks	−0.830
Sweetened beverages	−0.004
Other drinks	−0.476

**Change in body weight (kg)**			
	**Mean**	**Min**	**Max**
1st quintile	−0.89	−5.01	0.17
Sample mean	−1.72	−8.52	0.00
5th quintile	−2.11	−9.65	0.00

**Change in daily energy intake (kJ)**			
	**Mean**	**Min**	**Max**
1st quintile	−179.045	−1001.83	34.55121
Sample mean	−342.487	−1704.23	0
5th quintile	−427.682	−1943.13	0

*Note:* The left table shows percentage variations in quantities purchased per month 1 year after the introduction of the fat tax. No variation is detected for Cereals and Rice, Poultry, Alcohol and Food away from home. The right table shows changes in body weight (kg) and daily energy intake (kJ) 1 year after the introduction of the fat tax.

To convert weight loss into monetary benefits, we use a two‐part model (2 p.m.) of monthly health expenditures (Jones 2000), as adopted by Cawley and Meyerhoefer (2012). The first part estimates the probability of positive health expenditure using a Probit model, while the second part models the amount spent, if applicable, using a Generalized Linear Model (GLM). Monthly health expenditure data from the HBS includes costs for general practitioners, specialists, dentists, paramedical services, diagnostic tests, hospitalization, prescription and non‐prescription drugs, and other health‐related products. Expenditures vary by income, with the lowest income quintile spending 20% less than the highest. Despite Italy's national healthcare system, high‐income individuals may prefer private specialists to avoid long waiting times, resulting in higher health expenditures for them. We use statistical matching (Alpman 2016) to match health expenditures in the HBS with on weight, height and BMI from the 2015 Italian module of the European Health Interview Survey (EHIS).^24^ Our base regression specification for estimating the marginal impact of weight on health expenditures is:

(6) $$he_{i}=\alpha +\beta^{\prime }X_{i}+\varepsilon_{i}$$

where $he_{i}$ denotes monthly health expenditures (in Euro) by household *i*; α is the constant term and $X_{i}$ denotes a vector of explanatory variables including, age, gender, education level, employment position, marital status, macro‐region, income quintile, weight (kg) and height (cm) of each individual; $\varepsilon_{i}$ is the idiosyncratic error term. Table 5 shows regression results for the sample resulting from the matching. The marginal effects of interest are the GLM coefficients. The first two columns show average Probit coefficients and GLM coefficients (marginal effects), respectively. Columns 3 and 4 and 5 and 6 show Probit and GLM coefficients for the first and fifth quintile of the expenditure distribution. The GLM coefficient of weight for individuals in the first quintile of the expenditure distribution is negative, but not statistically significant at any significance level. So, weighing one more kilogram does not raise health expenditure for this group of individuals. Instead, one additional kg of weight increases health expenditures by almost 4€ per month on average, and by 6€ per month for individuals in the fifth quintile of the expenditure distribution. Conversely, losing 1 kg decreases monthly health expenditure by the same amounts. We acknowledge two limitations of our estimates. First, numerous studies, including Cawley and Meyerhoefer (2012), suggest that the coefficients may be biased due to measurement error from self‐reported rather than directly measured weight, with an ambiguous direction of bias stemming from reporting error. For instance, heavier individuals may tend to underreport their weight more. Another source of reporting error occurs when information is provided by a single household member, who may not accurately report the weight of others in the household (Burkhauser and Cawley 2008). Second, our study measures the correlation of weight with medical care costs, rather than the causal effect of weight on these costs. Regarding the first limitation, our weight data is from the 2015 Italian module of the European Health Interview Survey (EHIS), and it is self‐reported. However, since our sample consists solely of one‐member households, the second source of measurement error mentioned above does not apply. As for endogeneity in weight, reliance on a matched dataset limits our access to valid instrumental variables, such as, for example, the weight of a biological relative, which was employed by Cawley and Meyerhoefer (2012) to address these limitations.^25^

**TABLE 5:** Marginal effects of weight on monthly health expenditures.

Health expenditures (Dep. Var.)	Sample average		1st quintile		5th quintile	
	Probit	GLM	Probit	GLM	Probit	GLM
Weight (kg)	0.0157** (0.00758)	3.979*** (1.398)	0.0209*** (0.00564)	−0.325 (0.616)	0.0115 (0.00878)	5.965*** (1.924)
Height (cm)	0.0113* (0.00669)	5.098*** (1.424)	0.00217 (0.00844)	2.086** (0.876)	0.00723 (0.00961)	8.186*** (2.574)
Gender (1 = male)	0.791*** (0.178)	153.0*** (34.92)	0.735*** (0.140)	18.40 (18.46)	0.729*** (0.241)	250.6*** (52.54)
Age	0.0815*** (0.00876)	12.43*** (2.287)	0.0562** (0.0274)	8.583*** (3.006)	0.0851*** (0.0170)	17.81*** (5.428)
NorthEast	−0.00255 (0.0479)	10.63 (10.48)	0.180 (0.193)	49.20** (21.82)	−0.0437 (0.0783)	−3.196 (23.54)
Center	−0.0368 (0.0456)	−7.103 (10.83)	0.121 (0.157)	−0.999 (18.51)	−0.0832 (0.0837)	−24.17 (25.64)
South	0.279*** (0.0447)	−29.58*** (11.34)	0.494*** (0.131)	−7.732 (14.85)	−0.0134 (0.0962)	−64.31** (31.57)
Islands	0.0964 (0.0773)	−19.78 (21.53)	0.211 (0.185)	−33.94 (21.54)	−0.157 (0.176)	−97.27* (57.55)
2nd Quintile	0.347*** (0.0708)	21.18 (20.20)				
3rd Quintile	0.704*** (0.0828)	45.78** (18.77)				
4th Quintile	1.067*** (0.0774)	109.8*** (19.13)				
5th Quintile	1.380*** (0.0643)	231.0*** (19.51)				
Education	−0.0656*** (0.0253)	−11.45* (6.538)	−0.152* (0.0853)	1.231 (8.835)	−0.0431 (0.0485)	−21.88 (15.34)
Marital status	−0.0298** (0.0144)	−11.10*** (3.667)	−0.0118 (0.0445)	−10.94* (5.590)	−0.0305 (0.0247)	−12.36 (7.596)
Employment position	−0.0332 (0.0452)	−12.92 (10.15)	−0.0571 (0.0946)	−7.305 (10.03)	0.0822 (0.0916)	27.07 (27.66)
Constant	−5.069*** (1.804)	−1335*** (369.0)	−3.467** (1.470)	−323.8* (166.0)	−2.762 (2.306)	−1937*** (557.2)
Obs	8513	8513	887	887	2585	2585

*Note:* Columns 1 and 2 show results of the Probit regression (first part of the two‐parts model) and of the GLM regression (second part of the two‐parts model) for the entire sample. Columns 3 and 4 and 5 and 6 show the results for the first and fifth quintile, respectively, of the expenditure distribution. * = *p* < 0.10; ** = *p* < 0.05; *** = *p* < 0.01. Standard errors in parentheses.

**FIGURE 5:**
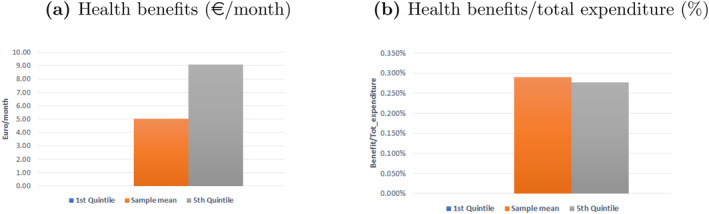
Health benefits. (a) shows health benefits, calculated as savings in health expenditures (€/month) due to weight lost after the tax, in €/month across the distribution of total expenditure, our proxy for disposable income. (b) shows health benefits as a fraction of total expenditure. No benefits emerge for individuals in the first quintile of the expenditure distribution as the marginal impact of weight on health expenditures is zero for this group.

As expected, individuals in the first quintile do not benefit from weight loss, as their health expenditure is significantly lower than individuals in the highest quintile.^26^ To obtain the monetary value of health benefits we multiply the vector of marginal effects in Table 5 by the vector of weight variations resulting from the tax (right hand side of Table 4). Benefits (measured as reduction in monthly health expenditure across different total expenditure groups) are shown in Figure 5 in €/month (Figure 5 a) and as a fraction of total consumption expenditure (Figure 5 b). Since the GLM weight coefficient for individuals in the first quintile of the expenditure distribution is not statistically significantly different from zero, no benefits emerge for low‐income individuals. High‐income individuals benefit more than individuals at the sample mean of the expenditure distribution. Relative to income, we do not detect remarkable differences between individuals at the sample mean of the expenditure distribution and individuals in the highest quintile of the expenditure distribution.

### 5.1 Net Welfare Impacts

**FIGURE 6:**
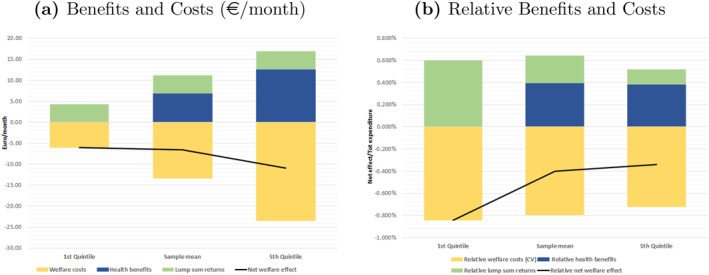
Net Welfare Impacts. (a) decomposes welfare changes resulting from the fat tax across the distribution of total expenditure. “Welfare costs” are measured by the compensating variation (€/month). “Health benefits” are calculated as savings in health expenditures (€/month) due to weight lost after the tax. “Lump sum return” is public revenues (€/month) from the fat tax redistributed equally across the distribution of total expenditure. The bold black line is the “Net welfare effect”, that is the difference between “Welfare costs” and “Health benefits”. (b) decomposes costs, benefits and net impacts relative to total expenditure.

We next combine the welfare costs with the estimated monetary benefits of weight loss to compute the net welfare impacts of the fat tax. Welfare effects are decomposed into three distinct components. They are plotted in Figure 6 across the distribution of total expenditure. “Redistributed Revenues” are public revenues from the fat tax equally redistributed as lump‐sum transfers. “Welfare benefit” is the money‐metric welfare benefit due to weight loss. “Welfare cost” is the compensating variation, that is the amount of money that makes the choice between an increase in their income or the introduction of the tax indifferent to consumers. “Net Welfare Impact” is the difference between welfare costs and benefits. Figure 6 shows that welfare costs are higher than benefits for all groups. In addition, net impacts result in small and progressive net losses. This is different from the results of Allcott, Lockwood, and Taubinsky (2019a), who found, in the context of a sugar tax, small and regressive net benefits. The lump sum returns only marginally offset the welfare costs of the tax. The right hand side of Figure 6 shows costs and benefits as a fraction of total expenditure, our proxy for income. Relative to total expenditure, the fat tax generates small and regressively distributed net losses, in line with Allcott, Lockwood, and Taubinsky (2019a).

We also simulate an alternative *ad valorem* tax reducing consumption of saturated fat by 30% and resulting in an increase in the price of food categories high in saturated fats: processed meat, snacks and sweets and fat and cheese. Results are shown in Supporting Information S1: Appendix F. Again, benefits are lower than costs for all groups. Compared to the specific tax, ad valorem taxation implies slightly smaller benefits for individuals in the highest quintile of the expenditure distribution but the net welfare impacts of the two policies are similar in magnitude.

## 6 Summary and Conclusion

Excise taxes are commonly used to mitigate socially costly consumption habits, but while welfare costs of new tax policies are often examined, the net welfare impacts—including potential benefits—are rarely assessed. This gap in the literature is important, as considering benefits can enhance the social and political acceptability of such policies. Our study fills this gap by evaluating the net welfare effects of taxes on unhealthy foods, specifically focusing on a fat tax in Italy. We examine both the costs and short‐term benefits, proxied by the monetary value of weight loss.

First, we assess the appropriateness of a nutrient‐based tax (on sugar or saturated fat) for Italian consumers. While sugar consumption for both singles and families is near the WHO threshold of 30 g/day, saturated fat consumption significantly exceeds this limit. Thus, a fat tax is more suitable for Italy. Second, we estimate the costs of taxation in terms of compensating variation. Our results show that high‐income individuals are likely to reduce fat consumption more in response to the tax, resulting in a larger direct consumer surplus loss for this group. To account for differences between singles and families with children, we estimate demand systems and elasticities for both groups. We find only minor differences in elasticities between single adults and families with one child, indicating similar welfare costs for both groups. Finally, to ensure consistency in expenditure and welfare impact, we focus on single adults to calculate the benefits of the proposed fat tax, proxied by weight reduction effects. Contrary to expectations, our analysis reveals progressive net welfare losses, especially among high‐income individuals.

One limitation of our study is the exclusive focus on weight gain from excessive saturated fat consumption, excluding broader health impacts and potential savings in public healthcare costs. Additionally, we do not address child obesity, a significant issue in Italy, although recent research suggests that broader policy approaches may be more effective for this problem (Crudu, Neri, and Tiezzi 2021). Despite these limitations, we hope our study contributes to shifting the discussion of “sin taxes” from a narrow focus on welfare costs to a more comprehensive assessment, aiding policymakers in making more informed decisions.

## Conflicts of Interest

The authors declare no conflicts of interest.

## Supporting information

Supporting Information S1

## Data Availability

The data that support the findings of this study are available from the corresponding author upon reasonable request.

## Endnotes

^1^
An exception is Dubois, Griffith, and O’Connell (2020) who study purchase decisions made by individuals for immediate consumption on‐the‐go. In this study, purchases and consumption are closely aligned.

^2^
According to ISTAT, Annuario Statistico Italiano 2019, at January $1^{st}$ 2019 one member households accounted for the largest share of Italian households: 33%. https://www.istat.it/it/archivio/236772.

^3^
Public funding of Italy's SSN accounted for 74.2% of total health spending in 2018, with total expenditure standing at 8.8% of GDP (OECD 2019).

^4^

https://www.istat.it/it/archivio/180341.

^5^
ISTAT gathers data via a weekly diary for frequently purchased items and interviews for durable goods, classifying expenditures into around 280 elementary goods and services.

^6^
Samples are drawn independently according to a two‐stage design; more details are in the ISTAT File Standard‐Indagine sui Consumi delle Famiglie‐Manuale d'uso, 2014. Downloadable at http://www.istat.it/it/archivio/4021.

^7^
One limitation is that we lack detailed data on food‐away‐from‐home consumption, which constitutes 22% of total food expenditure in our sample, including food from bars, restaurants, and on‐the‐go (e.g., vending machines). We can, however, disaggregate this category into three major segments: on‐the‐go (7%), bars and pastry shops (0.6%), and restaurants (14.5%). Although on‐the‐go food may be significant, especially for children (Dubois, Griffith, and O’Connell 2020), it accounts for only 7.6% of total expenditure in our adult sample.

^8^
Supporting Information S1: Figure B1 in Appendix B shows the monthly log price indices for our 16 food groups.

^9^

http://www.bda‐ieo.it/wordpress/en/.

^10^
Nutrient values for food away from home were not calculated due to the lack of detailed meal composition data. However, estimated elasticities suggest minimal impact on our findings, as complementarity or substitution effects between food away from home and other food groups appear small.

^11^
Fletcher, Frisvold, and Tefft (2010) show that in the U.S., soft drink taxes moderately reduce children's soft drink consumption but lead to offsetting increases in other high‐calorie beverages.

^12^
The EASI demand system has several additional benefits with respect to the popular Quadratic Almost Ideal Demand (QAID) system (Banks, Blundell, and Lewbel 1997). First, it makes it possible to specify and test for Engel curves that are more flexible than quadratic ones. This is an important characteristic when estimating a highly disaggregated demand system such as the present, and it may have an impact on price coefficient estimates. Second, the EASI error term can be interpreted as unobserved consumer heterogeneity that is seldom explained by observed demographic and price changes alone. These unobserved preference heterogeneity parameters show up both in the budget‐share and cost functions, and are therefore relevant factors for predicting demand and assessing welfare variations after a price change.

^13^
Lewbel and Pendakur (2009) show that the linearized version of the model estimated by OLS performs almost as well as fully‐efficient nonlinear estimation.

^14^
The vector $z_{k}$ includes the following variables: a dummy for gender (1 = male); the level of education in 5 classes (1 = no formal education, 2 = primary school, 3 = lower middle school, 4 = high school diploma, 5 = undergraduate or postgraduate degree); marital status in 6 classes (1 = single, 2 = married, 3 = married but not co‐habiting, 4 = legally separated, 5 = divorced, 6 = widowed); employment status in 7 classes (1 = employed, 2 = in search of first employment, 3 = unemployed, 4 = student, 5 = housewife, 6 = other employment position, 7 = retired); age in 9 classes (1 = between 18 and 24 years, 2 = between 25 and 29 years, 3 = between 30 and 34 years, 4 = between 35 and 39 years, 5 = between 40 and 44 years, 6 = between 45 and 49 years, 7 = between 50 and 54 years, 8 = between 55 and 59 years, 9 = between 60 and 64 years); three Census regions (metropolitan area, medium size city, small town).

^15^
We estimated the model twice: once for the sample of single adults and once for the sample of two adults and one child. Estimated coefficients for both models are available from the authors upon request.

^16^
Supporting Information S1: Appendix D provides equations for Marshallian price and expenditure elasticities and Hicksian price elasticities derived from the EASI demand model. Standard errors are bootstrapped with 200 replications.

^17^
Studies like Allcott, Lockwood, and Taubinsky (2019a), focusing on soft drinks alone, find higher elasticities (−1.37).

^18^
Full sets of uncompensated (Marshallian) elasticities and standard errors are available in Supporting Information S1: Table D1 in Appendix D.

^19^
Griffith et al. (2019) review the pass‐through of soft‐drink taxes to prices finding that a 100% pass‐through is the most common finding. Dubois, Griffith, and O’Connell (2020), study the on‐the‐go segment of the UK market and add to the previous evidence suggesting a soda tax pass‐through close to 100%.

^20^
We also separately simulate an easy‐to‐implement and administer increase in the existing Value Added Tax (VAT) on the food groups richest in saturated fat: fat and cheese, processed meat and sweets and snacks. Current VAT on food products in Italy is 4% for necessities (vegetables, fruit, bread and pasta, fat and cheese and oil) and at 10% for non‐necessities (cereals and rice, meat, fish, sweet and snacks, sweetened beverages and other beverages). The results of this additional counterfactual experiment are shown in Supporting Information S1: Appendix F.

^21^
In the context of a sugar tax in Catalonia, Fichera et al. (2021) also find that the sugar tax has a stronger impact on wealthier consumers.

^22^
Results for two adults and one child are not reported to save space. They do not markedly differ from those depicted in Figure 4. The compensating variation exhibits less variability across total expenditure compared to single adults, yet both absolute and relative CV patterns remain similar. At mean income, the absolute CV stands at 11.53€per month and 18.49€per month for the fifth quintile. As a percentage of total expenditure, the CV shows less variability compared to the same metric calculated for singles, with values of 0.47%, 0.43%, and 0.38% for the first, mean, and fifth quintiles respectively.

^23^
We thank one reviewer for suggesting this.

^24^
Supporting Information S1: Table E2 in Appendix E shows descriptive statistics for the matched variables used in our analysis.

^25^
Another potential limitation of our estimates of the monetary value of weight loss is that they are based on a selected sample of single‐member households, which may result in unknown biases. For instance, individuals with chronic diseases or injuries, who typically incur higher medical costs, may be less likely to live alone compared to healthier individuals. Conversely, individuals from poorer households with lower medical costs may not be able to afford living independently.

^26^
Interestingly, in the first‐step probit regression, the weight coefficient is positive and significant at 5% for the first quintile, with an implied elasticity close to 0.7. So gaining an extra kilogram increases the probability of positive health expenditure for individuals in the lowest quintile, even if they do not benefit from a one‐kg reduction when their health expenditure is already positive.
