# Supplementary material for: Fat vs. Sugar: The Case for a Saturated Fat Tax in Italy
Source: Health Econ. 2025 Jan 12;34(4):727–40. doi: 10.1002/hec.4933 (PMC11890089; doi:10.1002/hec.4933)
Supplement: Supplementary file 1 — Supporting Information S1 [file HEC-34-727-s001.docx]

Online appendix for

Sugar vs. Fat: The Case for a Saturated Fat Tax in Italy

## 12th December 2024

**Abstract**

This supplement contains plots of excess consumption of sugar and saturated fat for households of different size (section A); descriptive statistics (section B), Engel curve plots for the 16 food groups (section C), additional estimated elasticities (section D), the details of the statistical matching procedure (section E), and an additional counterfactual experiment considering a Value Added Tax (VAT) on fat and cheese, processed meat and sweets and snacks (section F).

*Keywords*: unhealthy food taxes, welfare benefits, welfare costs, exact affine stone index demand system, demand elasticities, micronutrients intake.

*JEL classification*: O12, D12, I15.

**Contents**

1. [Excess consumption of sugar and fat](#_bookmark1) 2
2. [Descriptive Statistics](#_bookmark2) 3
3. [Engel curves](#_bookmark3) 11
4. [Elasticities](#_bookmark4) 12
5. [Statistical Matching](#_bookmark5) 17
6. [Ad valorem tax](#_bookmark7) 20

# Excess consumption of sugar and fat

In this appendix we use data on food consumption expenditure in the Household Budget Survey (ISTAT) combined with nutrients data from the European Institute of Oncology (EIO) Composition Database for Epidemiological Studies (CDES) in Italy. Figure 1 doc- uments excess consumption of added sugar and saturated fats in households with more than one member (i.e. two adults, two adults and one child, two adults and two children) across income quintiles. For each household type, excess consumption of added sugar and saturated fats increases along the distribution of income, consistently with what we observe for singles. However, no noticeable excess consumption of saturated fats exceed that of sugar for all hosehold types.

**Figure 1:** Consumption of sugar and saturated fats in households of different sizes

**(a)** Two adults **(b)** Two adults, two children


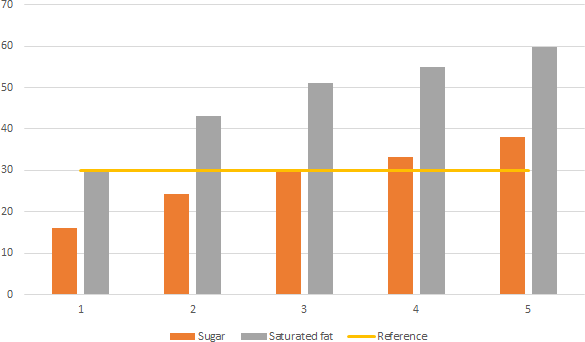

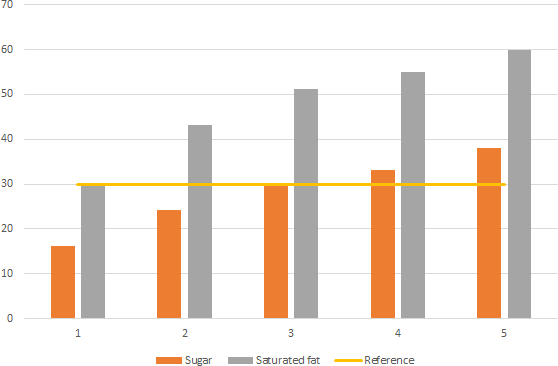


**Notes**: Average daily intake (grams/day) of sugar and saturated fats for equivalent adult in households with two adults and one child less than 14 years old (a) and for equivalent adult in households with two adults and two children less than 14 years of age (b) across quintiles of total expenditure. Horizontal lines are WHO recommended thresholds (30 grams/day).

# Descriptive Statistics

**Table B1:** Summary Statistics

**Expenditure Shares**

**One adult Two adults & one child**

|  | Obs. | Mean | Std. Dev. | Min | Max | Obs. | Mean | Std. Dev. | Min | Max |  |
| --- | --- | --- | --- | --- | --- | --- | --- | --- | --- | --- | --- |
| Alcohol | 12,369 | 0.010 | 0.018 | 0 | 0.223 | 4,772 | 0.007 | 0.011 | 0 | 0.216 |  |
| Bread & Pasta | 12,369 | 0.013 | 0.013 | 0 | 0.204 | 4,772 | 0.014 | 0.011 | 0 | 0.144 |  |
| Cereals & Rice | 12,369 | 0.003 | 0.005 | 0 | 0.092 | 4,772 | 0.003 | 0.004 | 0 | 0.068 |  |
| Eggs & Milk | 12,369 | 0.010 | 0.010 | 0 | 0.133 | 4,772 | 0.014 | 0.012 | 0 | 0.146 |  |
| Fat & Cheese | 12,369 | 0.012 | 0.012 | 0 | 0.176 | 4,772 | 0.014 | 0.010 | 0 | 0.177 |  |
| Fish | 12,369 | 0.013 | 0.018 | 0 | 0.278 | 4,772 | 0.015 | 0.017 | 0 | 0.147 |  |
| Food-away-from-home | 12,369 | 0.051 | 0.064 | 0 | 0.671 | 4,772 | 0.046 | 0.050 | 0 | 0.401 |  |
| Fruit | 12,369 | 0.015 | 0.014 | 0 | 0.183 | 4,772 | 0.015 | 0.012 | 0 | 0.135 |  |
| Oil | 12,369 | 0.005 | 0.009 | 0 | 0.243 | 4,772 | 0.004 | 0.008 | 0 | 0.213 |  |
| Other | 12,369 | 0.816 | 0.105 | 0.240 | 1.000 | 4,772 | 0.806 | 0.093 | 0.333 | 1.000 |  |
| Otherdrinks | 12,369 | 0.010 | 0.010 | 0 | 0.309 | 4,772 | 0.010 | 0.009 | 0 | 0.100 |  |
| Processed Meat | 12,369 | 0.012 | 0.014 | 0 | 0.159 | 4,772 | 0.014 | 0.011 | 0 | 0.098 |  |
| Poultry | 12,369 | 0.007 | 0.010 | 0 | 0.145 | 4,772 | 0.008 | 0.010 | 0 | 0.125 |  |
| Red meat | 12,369 | 0.014 | 0.018 | 0 | 0.203 | 4,772 | 0.016 | 0.017 | 0 | 0.172 |  |
| Sweet drinks | 12,369 | 0.004 | 0.006 | 0 | 0.102 | 4,772 | 0.005 | 0.005 | 0 | 0.068 |  |
| Sweets & Snacks | 12,369 | 0.017 | 0.015 | 0 | 0.180 | 4,772 | 0.021 | 0.014 | 0 | 0.140 |  |
| Vegetables | 12,369 | 0.023 | 0.021 | 0 | 0.267 | 4,772 | 0.022 | 0.017 | 0 | 0.143 |  |

**Log prices**

**One adult Two adults & one child**

|  | Obs. | Mean | Std. Dev. | Min | Max | Obs. | Mean | Std. Dev. | Min | Max |  |
| --- | --- | --- | --- | --- | --- | --- | --- | --- | --- | --- | --- |
| Alcohol | 12,369 | -0.658 | 0.271 | -1.609 | 0.889 | 4,772 |  |  |  |  |  |
| Bread & Pasta | 12,369 | -1.730 | 0.168 | -2.280 | -1.426 | 4,772 | -1.114 | 0.190 | -1.757 | -0.675 |  |
| Cereals & Rice | 12,369 | -3.437 | 0.272 | -4.256 | -2.773 | 4,772 | -2.843 | 0.283 | -3.660 | -2.117 |  |
| Eggs & Milk | 12,369 | -1.670 | 0.188 | -2.867 | 1.240 | 4,772 | -0.953 | 0.192 | -2.038 | -0.551 |  |
| Fat & Cheese | 12,369 | -0.818 | 0.177 | -1.686 | -0.276 | 4,772 | -0.418 | 0.197 | -1.148 | 0.183 |  |
| Fish | 12,369 | -1.190 | 0.227 | -1.991 | -0.609 | 4,772 | -0.519 | 0.216 | -1.372 | 0.144 |  |
| Food-away-from-home | 12,369 | 0.191 | 0.229 | -0.797 | 0.826 | 4,772 | 0.439 | 0.296 | -0.669 | 1.276 |  |
| Fruit | 12,369 | -0.400 | 0.243 | -1.589 | 0.112 | 4,772 | -0.098 | 0.263 | -1.115 | 0.629 |  |
| Oil | 12,369 | 2.658 | 0.309 | -3.636 | -1.568 | 4,772 |  |  |  |  |  |
| Other | 12,369 | 3.075 | 0.269 | 1.737 | 4.114 | 4,772 | 3.213 | 0.237 | 2.147 | 3.912 |  |
| Otherdrinks | 12,369 | -1.996 | 0.192 | -2.639 | -1.596 | 4,772 | -1.468 | 0.195 | -1.985 | -1.032 |  |
| Processed Meat | 12,369 | -1.472 | 0.169 | -2.037 | -0.967 | 4,772 | -0.883 | 0.166 | -1.428 | -0.504 |  |
| Poultry | 12,369 | 0.010 | 0.013 | -0.004 | 0.038 | 4,772 | -1.389 | 0.172 | -2.000 | -0.697 |  |
| Red meat | 12,369 | -1.162 | 0.186 | -1.980 | -0.688 | 4,772 | -0.728 | 0.179 | -1.274 | -0.288 |  |
| Sweet drinks | 12,369 | -2.779 | 0.253 | -3.494 | -1.957 | 4,772 | -1.983 | 0.257 | -2.688 | -1.389 |  |
| Sweets & Snacks | 12,369 | 0.710 | 0.315 | -1.026 | 1.391 | 4,772 | 0.870 | 0.315 | -0.328 | 1.886 |  |
| Vegetables | 12,369 | 1.623 | 0.534 | -0.757 | 3.048 | 4,772 | 1.547 | 0.497 | -0.409 | 3.171 |  |

**Notes:** Expenditure shares computed using total current monthly expenditure at the denominator for both household types (upper panel). Summary statistics of logs of Lewbel prices for both household types (lower panel). The logs of Alcohol and Poultry Lewbel prices for two adults and one child are not reported due to Lewbel procedure not converging for these items

**Table B2:** Summary Statistics cont’ed

**Control Variables**

**One adult Two adults & one child**

|  | Obs. | Mean | Std. Dev. | Min | Max | Obs. | Mean | Std. Dev. | Min | Max |  |
| --- | --- | --- | --- | --- | --- | --- | --- | --- | --- | --- | --- |
| Total monthly expenditure | 12,369 | 1869,209 | 1090.125 | 110 | 9697.53 | 4,772 | 2894.956 | 1421.251 | 326.6 | 9670.06 |  |
| Gender | 12,369 | 1.465 | 0.499 | 1 | 2 | 4,772 | 1.233 | 0.423 | 1 | 2 |  |
| Education | 12,369 | 3.831 | 0.828 | 1 | 5 | 4,772 | 3.925 | 0.744 | 1 | 5 |  |
| Marital status | 12,369 | 2.315 | 1.786 | 1 | 6 | 4,772 | 1.908 | 0.622 | 1 | 6 |  |
| Employment position | 12,369 | 2.011 | 1.971 | 1 | 8 | 4,656 | 1.924 | 1.004 | 1 | 4 |  |
| Age | 12,369 | 6.008 | 2.209 | 1 | 9 | 4,772 | 7.800 | 1.546 | 4 | 15 |  |
| Metropolitan area | 12,369 | 0.154 | 0.361 | 0 | 1 | 4,772 | 0.129 | 0.336 | 0 | 1 |  |
| Medium city | 12,369 | 0.294 | 0.456 | 0 | 1 | 4,772 | 0.272 | 0.445 | 0 | 1 |  |
| Small city | 12,369 | 0.551 | 0.497 | 0 | 1 | 4,772 | 0.599 | 0.490 | 0 | 1 |  |

**Notes:** Gender (1= male); Education (1= no formal education, 2= primary school, 3= lower middle school, 4= high school diploma, 5= undergraduate or postgraduate degree); Marital status (1= single, 2=married, 3= married but not co-habiting, 4= legally separ- ated, 5= divorced, 6 = widowed); Employment position (1= employed, 2= in search of first employment, 3=unemployed, 4= student, 5= housewife, 7= other employment position, 8= retired); Age(1= between 18 and 24 years, 2= between 25 and 29 years, 3= between

30 and 34 years, 4 = between 35 and 39 years, 5= between 40 and 44 years, 6=between 45 and 49 years, 7 = between 50 and 54 years,

8 = between 55 and 59 years, 9= between 60 and 64 years).

**Table B3:** Share of food expenditures by education level of the reference person.

**One adult**

| **Food groups** | no education | primary  school | lower  middle school | high  school | undergraduate  or postgrad. degree |
| --- | --- | --- | --- | --- | --- |
| Alcohol | 0.013 | 0.01 | 0.012 | 0.010 | 0.009 |
| Bread & pasta | 0.022 | 0.014 | 0.011 | 0.008 | 0.006 |
| Cereals & rice | 0.008 | 0.020 | 0.016 | 0.013 | 0.010 |
| Eggs & Milk | 0.017 | 0.014 | 0.012 | 0.01 | 0.008 |
| Fat & Cheese | 0.015 | 0.017 | 0.014 | 0.012 | 0.01 |
| Fish | 0.015 | 0.015 | 0.013 | 0.012 | 0.011 |
| Food-away-from-home | 0.024 | 0.021 | 0.041 | 0.05 | 0.064 |
| Fruit | 0.018 | 0.020 | 0.017 | 0.015 | 0.012 |
| Oil | 0.006 | 0.007 | 0.005 | 0.004 | 0.003 |
| Other | 0.740 | 0.754 | 0.788 | 0.822 | 0.859 |
| Otherdrinks | 0.014 | 0.013 | 0.011 | 0.01 | 0.008 |
| Processed meat | 0.014 | 0.017 | 0.015 | 0.004 | 0.009 |
| Poultry | 0.016 | 0.012 | 0.009 | 0.012 | 0.005 |
| Red meat | 0.023 | 0.022 | 0.017 | 0.007 | 0.01 |
| Sweet drinks | 0.003 | 0.005 | 0.004 | 0.014 | 0.003 |
| Sweets & snacks | 0.003 | 0.018 | 0.018 | 0.004 | 0.015 |
| Vegetables | 0.003 | 0.034 | 0.026 | 0.022 | 0.018 |
| Obs. | 83 | 526 | 3,387 | 5,781 | 2,592 |
|  | **Two** | **adults &** | **one child** |  |  |
| **Food groups** | no education | primary | lower | high | undergraduate |
|  |  | school | middle school | school | or postgrad. degree |
| Alcohol | 0.013 | 0.007 | 0.008 | 0.008 | 0.007 |
| Bread & pasta | 0.019 | 0.025 | 0.017 | 0.014 | 0.011 |
| Cereals & rice | 0.003 | 0.005 | 0.003 | 0.003 | 0.002 |
| Eggs & Milk | 0.009 | 0.019 | 0.016 | 0.013 | 0.021 |
| Fat & Cheese | 0.009 | 0.015 | 0.014 | 0.014 | 0.012 |
| Fish | 0.010 | 0.016 | 0.015 | 0.015 | 0.014 |
| Food-away-from-home | 0.039 | 0.014 | 0.035 | 0.046 | 0.060 |
| Fruit | 0.013 | 0.021 | 0.016 | 0.015 | 0.014 |
| Oil | 0.002 | 0.008 | 0.005 | 0.004 | 0.003 |
| Other | 0.805 | 0.736 | 0.780 | 0.807 | 0.839 |
| Otherdrinks | 0.004 | 0.007 | 0.011 | 0.009 | 0.008 |
| Processed meat | 0.011 | 0.017 | 0.016 | 0.014 | 0.010 |
| Poultry | 0.005 | 0.015 | 0.010 | 0.008 | 0.006 |
| Red meat | 0.019 | 0.025 | 0.019 | 0.016 | 0.012 |
| Sweet drinks | 0.006 | 0.007 | 0.006 | 0.005 | 0.004 |
| Sweets & snacks | 0.016 | 0.026 | 0.022 | 0.021 | 0.018 |
| Vegetables | 0.018 | 0.031 | 0.025 | 0.022 | 0.019 |
| Obs. | 6 | 71 | 1,264 | 2,366 | 1,065 |

**Notes**: For both household types, expenditure shares are computed using total current monthly expenditure at the denominator.

**Table B4:** Share of food expenditures by geographic area.

**One adult Two adults & one child**

**Food groups**

North Centre South Islands North Centre South Islands

Alcohol 0.011 0.009 0.011 0.009

Bread & pasta 0.011 0.014 0.016 0.017

Cereals & rice 0.002 0.003 0.004 0.003

Eggs & Milk 0.009 0.010 0.012 0.011

Fat & Cheese 0.012 0.012 0.014 0.011

Fish 0.009 0.013 0.016 0.019

Food-away-from-home 0.061 0.046 0.038 0.044

Fruit 0.013 0.016 0.017 0.017

Oil 0.004 0.004 0.005 0.006

Other 0.840 0.820 0.779 0.793

Otherdrinks 0.008 0.009 0.012 0.014

Processed meat 0.011 0.013 0.015 0.012

Poultry 0.006 0.007 0.010 0.08

Red meat 0.011 0.016 0.018 0.018

Sweet drinks 0.004 0.009 0.004 0.005

Sweets & snacks 0.016 0.015 0.019 0.016

Vegetables 0.020 0.024 0.027 0.025

0.008 0.007 0.007 0.007

0.013 0.014 0.017 0.019

0.003 0.003 0.003 0.003

0.012 0.013 0.017 0.017

0.013 0.012 0.016 0.013

0.012 0.016 0.019 0.019

0.053 0.049 0.034 0.031

0.014 0.015 0.017 0.016

0.004 0.004 0.005 0.006

0.827 0.813 0.770 0.774

0.008 0.010 0.011 0.013

0.013 0.014 0.016 0.015

0.007 0.009 0.010 0.009

0.014 0.016 0.020 0.020

0.005 0.005 0.005 0.007

0.020 0.019 0.023 0.022

0.020 0.022 0.025 0.023

Obs.

5,954 2,434 3,148

833 2,294

918 1,187

373

**Notes**: For both household types, expenditure shares are computed using total current monthly ex- penditure at the denominator.

**Table B5:** Share of food expenditures by gender of the reference person

### One adult Two adults & one child

| **Food groups** | Female | Male | Female | Male |  |
| --- | --- | --- | --- | --- | --- |
| Alcohol | 0.006 | 0.014 | 0.007 | 0.008 |  |
| Bread & pasta | 0.013 | 0.013 | 0.014 | 0.015 |  |
| Cereals & rice | 0.003 | 0.003 | 0.003 | 0.003 |  |
| Eggs & Milk | 0.011 | 0.009 | 0.013 | 0.014 |  |
| Fat & Cheese | 0.013 | 0.012 | 0.013 | 0.014 |  |
| Fish | 0.013 | 0.012 | 0.015 | 0.015 |  |
| Food-away-from-home | 0.036 | 0.064 | 0.048 | 0.045 |  |
| Fruit | 0.016 | 0.014 | 0.014 | 0.015 |  |
| Oil | 0.005 | 0.004 | 0.004 | 0.004 |  |
| Other | 0.820 | 0.815 | 0.816 | 0.803 |  |
| Otherdrinks | 0.010 | 0.009 | 0.009 | 0.010 |  |
| Processed meat | 0.012 | 0.013 | 0.013 | 0.014 |  |
| Poultry | 0.007 | 0.007 | 0.007 | 0.009 |  |
| Red meat | 0.014 | 0.015 | 0.015 | 0.017 |  |
| Sweet drinks | 0.004 | 0.004 | 0.005 | 0.005 |  |
| Sweets & snacks | 0.018 | 0.016 | 0.020 | 0.021 |  |
| Vegetables | 0.025 | 0.021 | 0.022 | 0.022 |  |
| Obs | 5,756 | 6,613 | 1,112 | 3,660 |  |

**Notes**: For both household types, expenditure shares are computed using total current monthly expenditure at the denominator.

**Table B6:** Share of food expenditures across the distribution of total expenditure

| **One adult** | | | |
| --- | --- | --- | --- |
| **Food groups** | 1*^st^* quintile | Central quintiles | 5*^th^* quintile |
| Alcohol | 0.009 | 0.010 | 0.011 |
| Bread & pasta | 0.021 | 0.013 | 0.007 |
| Cereals & rice | 0.004 | 0.003 | 0.002 |
| Eggs & Milk | 0.015 | 0.010 | 0.006 |
| Fat & Cheese | 0.015 | 0.013 | 0.009 |
| Fish | 0.013 | 0.013 | 0.011 |
| Food-away-from-home | 0.025 | 0.054 | 0.068 |
| Fruit | 0.018 | 0.016 | 0.012 |
| Oil | 0.005 | 0.005 | 0.004 |
| Other | 0.780 | 0.815 | 0.863 |
| Otherdrinks | 0.012 | 0.010 | 0.008 |
| Processed meat | 0.015 | 0.013 | 0.009 |
| Poultry | 0.012 | 0.008 | 0.005 |
| Red meat | 0.015 | 0.014 | 0.010 |
| Sweet drinks | 0.004 | 0.004 | 0.003 |
| Sweets & snacks | 0.018 | 0.017 | 0.014 |
| Vegetables | 0.029 | 0.023 | 0.017 |
| Obs | 2,474 | 7,421 | 2,474 |
| **Two adults and one child** | | | |
| **Food groups** | 1*^st^* quintile | Central quintiles | 5*^th^* quintile |
| Alcohol | 0.006 | 0.007 | 0.008 |
| Bread & pasta | 0.021 | 0.015 | 0.010 |
| Cereals & rice | 0.004 | 0.003 | 0.002 |
| Eggs & Milk | 0.019 | 0.014 | 0.010 |
| Fat & Cheese | 0.016 | 0.015 | 0.011 |
| Fish | 0.014 | 0.016 | 0.014 |
| Food-away-from-home | 0.020 | 0.044 | 0.061 |
| Fruit | 0.018 | 0.016 | 0.012 |
| Oil | 0.005 | 0.004 | 0.004 |
| Other | 0.767 | 0.790 | 0.841 |
| Otherdrinks | 0.011 | 0.010 | 0.008 |
| Processed meat | 0.016 | 0.016 | 0.011 |
| Poultry | 0.013 | 0.009 | 0.006 |
| Red meat | 0.020 | 0.018 | 0.013 |
| Sweet drinks | 0.006 | 0.005 | 0.003 |
| Sweets & snacks | 0.022 | 0.023 | 0.018 |
| Vegetables | 0.026 | 0.024 | 0.018 |
| Obs | 957 | 1,904 | 1,908 |

**Notes:** 1*^st^* quintile: between 110 and 1022 Euro/month; Central quintiles: between 1123 and 2542 Euro/month; 5*^th^* quintile: between 2543 and 9697 Euro/month.

**Figure B1:** Log monthly price indices (2014(1) - 2018(12))

115

110

Price Indices (2015=100)

105

100

95

Vegetables/Other_drinks

Fruit

Food_afh

Fish

Sweets_Snacks

Alcohol

Bread_Pasta

Cereals_rice

Eggs_Milk

Red_meat

Poultry

Processed_meat

Oil

Fat_Cheese

Sweet_drinks

0 20 40 60

Months

Source: ISTAT, Indice nazionale dei prezzi al consumo per l'intera collettività

**Notes:** The Figure shows that, for many food groups, the series of log monthly price indices displays little variation over time. This differential price variation over time coupled with no cross-sectional variation provides our motivation for using Lewbel prices.

# Engel curves

We estimate the demand system using seemingly unrelated regressions. Figures C1 and C2 plot single adults’ Engel curves for the 16 food groups using total current consumption expenditure as a proxy of available income. Inspection of these Figures suggests that the Engel curve shapes cannot be adequately represented by a linear or quadratic function. To determine the degree of the income polynomials, we add a degree at a time start- ing from *L* = 2 and test the joint significance of the *b_L_* coefficients by minimum distance [(Wooldridge,](#_bookmark12) [2010).](#_bookmark12) Under the null hypothesis that the *L^th^* degree of polynomial is exclud-

able, the test statistic is asymptotically distributed as *χ*^2^

(*J−*1)

. At *L* = 5 the test statistic

still rejects the null hypothesis. We therefore opted for a fifth polynomial in *y* to fit the Engel curves.

**Figure C1:** Kernel estimation of expenditure shares on log total expenditure


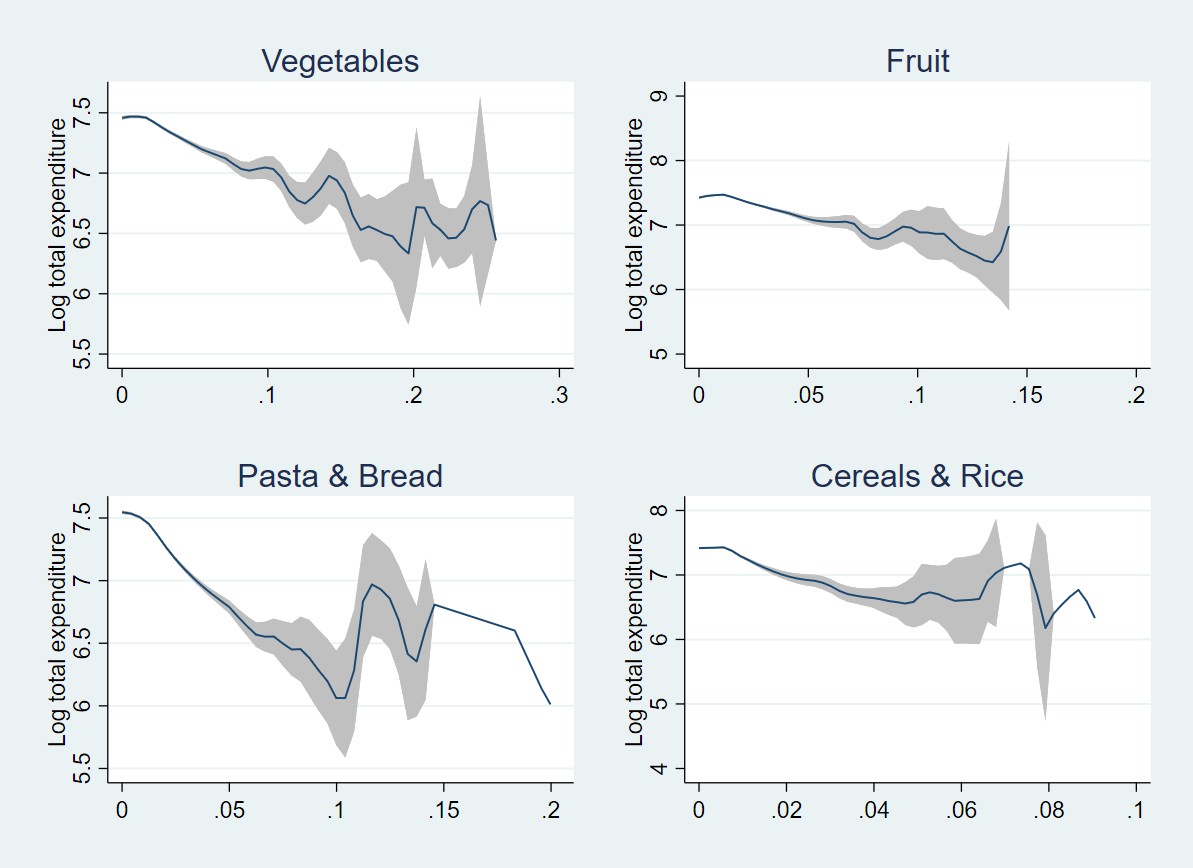

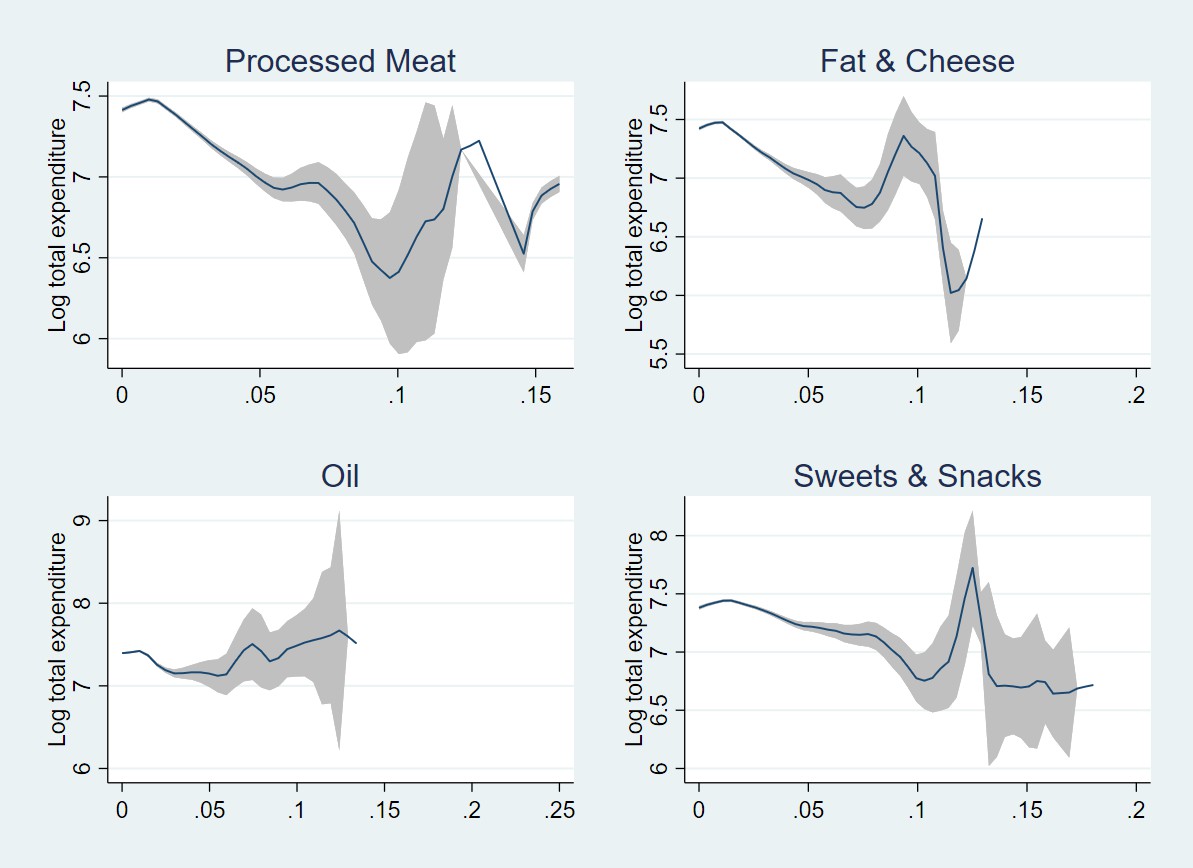

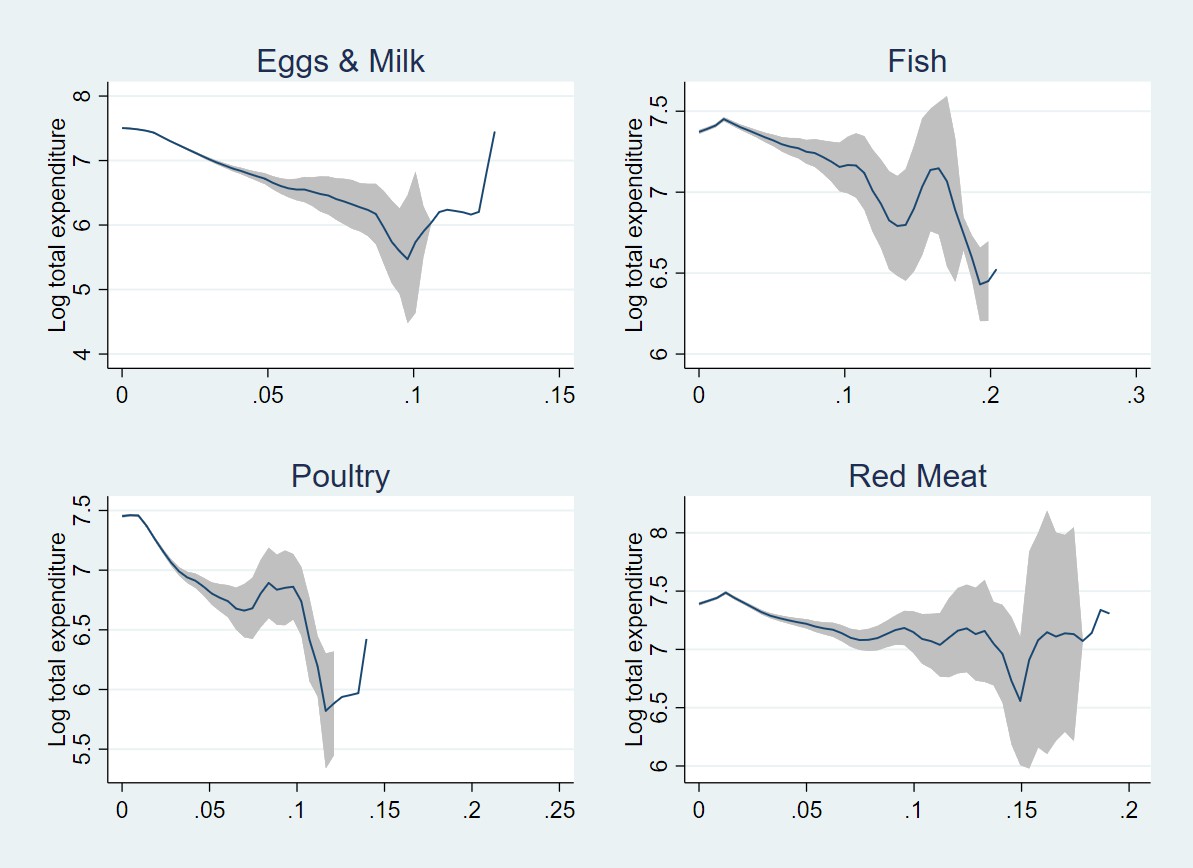

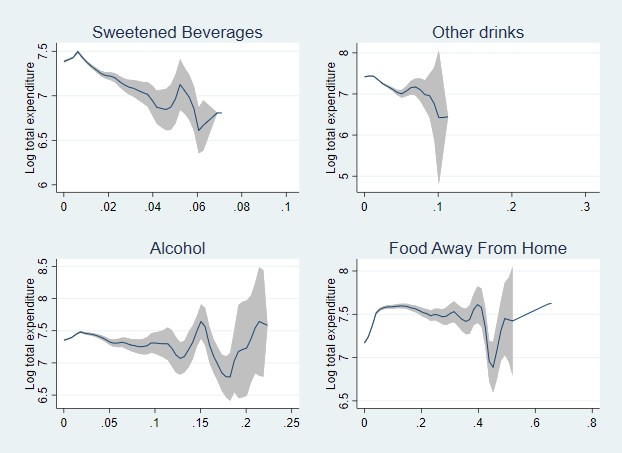


# Elasticities

EASI Marshallian and Hicksian price elasticities of quantities and expenditure elasticities are computed as [(Irz,](#_bookmark10) [2017)^[[1]](#footnote-1)^:](#_bookmark10)

$$\frac{\partial{lnq}^{i}}{\partial{lnp}^{j}}=\frac{\alpha^{ij}}{w^{i}}+\bar{w}^{j}-\delta_{ij}-w^{j}\left[ \sum_{r=1}^{R} b_{r}^{i}r\left( \hat{y} \right)^{r-1}+\frac{1}{w^{i}}+1 \right]$$

$$\frac{\partial{lnq}^{i}}{\partial lnx}=\frac{\alpha^{ij}}{w^{i}}+\bar{w}^{j}-\delta_{ij}-w^{j}\left[ \sum_{r=1}^{R} b_{r}^{i}r\left( \hat{y} \right)^{r-1} \right]+\frac{1}{w^{i}}+1$$

$$\left. \frac{\partial{lnq}^{i}}{\partial lnp^{j}} \right|_{\bar{u}}=\frac{\alpha^{ij}}{w^{i}}+\bar{w}^{j}-\delta_{ij}-w^{j}$$

13

**Table D1:** Uncompensated price elasticities (single adults) - sample means.

|  | Vegetables | Fruit | Pasta & bread | Cereals & Rice | Eggs & Milk | Fish | Poultry | Red meat | Processed meat | Fat & Cheese | Oil | Sweets & snacks | Sweetened beverages | Other drinks | Alcohol | Food-away-from-home |
| --- | --- | --- | --- | --- | --- | --- | --- | --- | --- | --- | --- | --- | --- | --- | --- | --- |
| Vegetables | **-1.906***** | -0.017 | 0.120*** | -0.003 | 0.082*** | -0.143*** | -0.003 | 0.073** | 0.043* | -0.005 | -0.035** | 0.185*** | -0.020** | 0.010 | -0.080*** | 0.066* |
| Fruit | -0.028 | **-2.203***** | 0.018 | -0.108*** | -0.013 | -0.042 | 0.001 | 0.045 | 0.106*** | -0.078** | -0.022 | 0.026 | 0.091*** | 0.022 | 0.146*** | 0.291*** |
| Pasta & bread | 0.215*** | 0.028 | **-0.781***** | -0.035 | -0.010 | -0.076 | -0.103* | 0.003 | -0.246*** | 0.084* | -0.006 | -0.055 | 0.005 | -0.019 | -0.050 | 0.132** |
| Cereals & and Rice | -0.003 | -0.558*** | -0.156 | **-0.165** | 0.114 | -0.076 | -0.087 | -0.256 | 0.207 | -0.017 | -0.190 | -0.411*** | -0.066 | -0.009 | 0.388** | 0.396* |
| Eggs & milk | 0.191*** | -0.014 | -0.016 | 0.031 | **-1.011***** | -0.141** | 0.048 | -0.236*** | -0.299*** | 0.056 | -0.040 | -0.151*** | 0.003 | 0.015 | 0.031 | 0.146* |
| Fish | -0.267*** | -0.056 | -0.091* | -0.021 | -0.118** | **-1.509***** | -0.017 | -0.055 | 0.049 | 0.003 | -0.048 | 0.051 | 0.043 | -0.058 | -0.086 | 0.090 |
| Poultry | 0.002 | 0.011 | -0.170* | -0.033 | 0.063 | -0.015 | **0.198** | -0.616*** | -0.092 | -0.145 | -0.247*** | 0.153* | -0.052 | 0.022 | -0.106 | -0.085 |
| Red meat | 0.120** | 0.050 | -0.004 | -0.058 | -0.180*** | -0.049 | -0.369*** | **-0.786***** | -0.176** | -0.083 | 0.046 | -0.028 | -0.023 | -0.114* | -0.174*** | -0.089 |
| Processed meat | 0.083* | 0.137*** | -0.267*** | 0.046 | -0.244*** | 0.058 | -0.062 | -0.187** | **-0.334***** | -0.108* | -0.058 | -0.076 | -0.014 | -0.241*** | -0.098 | 0.004 |
| Fat & cheese | -0.013 | -0.097** | 0.083* | -0.007 | 0.041 | 0.006 | -0.098* | -0.090 | -0.113* | **-1.447***** | 0.036 | -0.064 | -0.016 | -0.080 | -0.048 | 0.074 |
| Oil | -0.174** | -0.071 | -0.023 | -0.123 | -0.091 | -0.130 | -0.438*** | 0.141 | -0.161 | 0.100 | **-0.587***** | -0.072 | -0.164*** | 0.155 | -0.060 | 0.158 |
| Sweets & snacks | 0.232*** | 0.012 | -0.061* | -0.076*** | -0.102*** | 0.032 | 0.062 | -0.032 | -0.069 | -0.056 | -0.024 | **-3.801***** | -0.119*** | -0.125** | 0.103** | 0.459*** |
| Sweetened beverages | -0.112** | 0.350*** | 0.012 | -0.050 | 0.006 | 0.141* | -0.106 | -0.074 | -0.046 | -0.045 | -0.184*** | -0.478*** | **-0.809***** | -0.397*** | 0.035 | 0.244** |
| Otherdrinks | 0.032 | 0.043 | -0.025 | -0.004 | 0.017 | -0.065 | 0.017 | -0.149* | -0.300*** | -0.094 | 0.073 | -0.192** | -0.162*** | **-1.054***** | -0.059 | 0.161* |
| Alcohol | -0.168*** | 0.226*** | -0.065 | 0.107** | 0.032 | -0.096 | -0.083 | -0.221** | -0.116 | -0.051 | -0.025 | 0.189** | 0.015 | -0.056 | **-0.686***** | -0.408*** |
| Food-away-from-home | 0.029* | 0.089*** | 0.029* | 0.019* | 0.026* | 0.027 | -0.017 | -0.021 | -0.001 | 0.020 | 0.014 | 0.165*** | 0.019** | 0.027 | -0.086*** | **-2.822***** |

**Notes**: The cells of each row show the Marshallian price elasticity of the food group of the row due to a change in price of the food group of the column. For example, the third entry in the first column (0.228) is the percentage change in the demand for pasta and bread following a 1% increase in the price of vegetables.

∗ = *p <* 0*.*10; ∗∗ = *p <* 0*.*05; ∗ ∗ ∗ = *p <* 0*.*01. Bootstrapped standard errors with 200 replications in parentheses.

**Table D2:** Compensated own price and expenditure elasticities at different levels of total expenditure (single adults)

1*^st^* quintile 5*^th^* quintile

|  | Price | Expenditure | Price | Expenditure |
| --- | --- | --- | --- | --- |
| Vegetables | -2.566*** | 1.178*** | -1.651*** | 1.638*** |
| Fruit | -2.260*** | 1.709*** | -1.819*** | 1.348*** |
| Pasta & Bread | -0.866*** | 0.838*** | -0.768*** | 0.722*** |
| Cereals & Rice | 1.214* | -0.062 | -0.804*** | 1.618*** |
| Eggs & Milk | -0.822*** | 0.737* | -1.294*** | 1.052*** |
| Fish | -1.719*** | 1.862*** | -1.649*** | 1.476*** |
| Poultry | 0.128 | 0.733 | -0.704*** | 0.717** |
| Red Meat | -1.019*** | 2.027*** | -1.076*** | 0.915** |
| Processed Meat | -0.301 | 0.892 | -0.689*** | 0.926*** |
| Fat & Cheese | -0.938*** | 1.231*** | -1.561*** | 1.219*** |
| Oil | -0.324 | 1.103 | -1.004*** | 0.63 |
| Sweets & Snacks | -4.792*** | 2.128*** | -3.004*** | 2.591*** |
| Sweetened beverages | -1.172*** | 1.912** | -0.407*** | 0.316 |
| Other drinks | -0.781** | 0.695 | -1.240*** | 0.315 |
| Alcohol | 0.15 | -0.173 | -1.468*** | -0.448 |
| Food-away-from-home | -2.569*** | -0.179 | -2.361*** | -0.348 |
| Obs | 2474 | 2474 | 2474 | 2474 |

**Notes:** 1*^st^* quintile: between 110 and 1022 Euro/month; 5*^th^* quintile: between 2543 and 9697 Euro/month.

**Table D3:** Compensated own price elasticities by geographic area (single adults)

|  | North | Centre | South | Islands |
| --- | --- | --- | --- | --- |
| Alcohol | -1.386*** | -0.541* | 0.255 | -1.003* |
| Bread & pasta | -0.932*** | -0.548*** | -0.766*** | -0.587* |
| Cereals & rice | -0.794*** | 0.299 | 0.239 | -0.931 |
| Eggs & Milk | -1.044*** | -0.855*** | -0.906*** | -1.805*** |
| Fat & Cheese | -1.621*** | -0.993*** | -1.297*** | -1.545*** |
| Fish | -1.451*** | -1.674*** | -1.487*** | -1.352*** |
| Food-away-from-home | -2.974*** | -2.524*** | -2.471*** | -2.789** |
| Fruit | -2.215*** | -2.044*** | -2.149*** | -2.543*** |
| Oil | -0.888*** | -0.574** | -0.456** | 0.486 |
| Otherdrinks | -1.346*** | -0.481* | -0.995*** | -1.197* |
| Processed meat | -0.758*** | -0.746*** | 0.252 | 0.185 |
| Poultry | -0.306 | 0.362 | 0.575* | 0.844 |
| Red meat | -0.949*** | -0.780*** | -0.596*** | -0.373 |
| Sweetened beverages | -0.974*** | -0.604*** | -0.731*** | -0.739*** |
| Sweets & snacks | -3.944*** | -3.551*** | -3.716*** | -3.484*** |
| Vegetables | -1.887*** | -1.885*** | -2.081*** | -2.272*** |

**Notes:** ∗ = *p <* 0*.*10; ∗∗ = *p <* 0*.*05; ∗ ∗ ∗ = *p <* 0*.*01. Standard errors bootstrapped with 200 replications.

**Table D4:** Expenditure elasticities by geographic area (single adults)

|  | North | Centre | South | Islands |
| --- | --- | --- | --- | --- |
| Alcohol | 0.362** | 0.730* | 0.259 | 1.640** |
| Bread & pasta | 0.728*** | 0.641*** | 0.376** | 0.755** |
| Cereals & rice | 0.325 | -0.468 | 0.183 | 2.513* |
| Eggs & Milk | 0.688*** | 0.575*** | 0.771*** | 1.930*** |
| Fat & Cheese | 1.146*** | 1.012*** | 1.104*** | 1.282*** |
| Fish | 1.309*** | 1.228*** | 1.327*** | 2.342*** |
| Food-away-from-home | 0.761*** | 0.952*** | 1.012*** | 1.205** |
| Fruit | 1.172*** | 1.237*** | 0.979*** | 1.579*** |
| Oil | 1.074* | 0.294 | 1.206** | 0.953 |
| Otherdrinks | 1.015*** | 0.677* | 0.497 | 0.643 |
| Processed meat | 0.893*** | 0.690*** | 0.526** | 0.992* |
| Poultry | 0.635** | 0.369 | 0.463 | 0.098 |
| Red meat | 1.215*** | 1.050*** | 1.150*** | 1.26 |
| Sweetened beverages | 1.227** | 0.543 | 0.990*** | 0.903 |
| Sweets & snacks | 1.859*** | 1.839*** | 1.972*** | 2.581*** |
| Vegetables | 1.155*** | 0.858*** | 0.979*** | 1.123**** |

**Notes:** ∗ = *p <* 0*.*10; ∗∗ = *p <* 0*.*05; ∗ ∗ ∗ = *p <* 0*.*01. Standard errors boot- strapped with 200 replications.

# Statistical Matching

We adopt the two-step procedure outlined by [Alpman](#_bookmark9) [(2016)](#_bookmark9) to implement the statistical matching framework proposed by [Rubin](#_bookmark11) [(1986)](#_bookmark11) for integrating two datasets. Specifically, if Dataset 1 contains the variable weight and Dataset 2 contains health expenditures, both sharing a set of common variables, X, statistical matching facilitates the construction of a new dataset that includes weight, health expenditures, and X for all respondents.

Health expenditures are sourced from the 2015 Household Budget Survey (HBS), while individual weight is obtained from the 2015 European Health Interview Survey (EHIS). The shared variables across these datasets include: the number of family members, age, gender, income quintile, geographic location, education level, and employment status. The object- ive of the matching is to create a comprehensive dataset comprising health expenditures, individual weight, and control variables. This matched dataset is subsequently employed to estimate Equation (6) in our study.

The procedure consists of two steps. Prediction Step: predicted values for weight and health expenditures are generated for each observation in the incomplete datasets. These predictions are based on the assumed partial correlation between weight and health expenditures, conditional on the control variables. Matching Step: each observation in the EHIS lacking health expenditures is matched to the corresponding observation in the HBS with the closest predicted value of health expenditures (from Step 1), conditional on the control variables. Similarly, each observation in the HBS missing weight is matched to the EHIS observation with the closest predicted weight value, conditional on the same controls. The partial correlation (*ρ*) between health expenditures and weight, conditional on the control variables, is varied systematically between 0.1 and 1. To account for this variation, we perform multiple imputations using all values of *ρ* within this range. As recommended by [Alpman](#_bookmark9) [(2016),](#_bookmark9) multiple imputation mitigates the risk of downward bias in the estimated standard errors[^2^](#_bookmark0). For consistency with our main empirical analysis, we focus on single-person households aged under 65. Summary statistics for the initial and matched datasets are presented in Table [E1.](#_bookmark6)

As shown in Table [E2,](#_bookmark7) the original EHIS dataset includes 1,465 observations on in- dividual weight for respondents under 65. Using the Rubin procedure, 6,914 additional observations are matched with the HBS, resulting in a final dataset with 8,379 imputed weight values. Similarly, the original HBS dataset contains 12,419 observations on health expenditures, which increase to 13,593 after matching.

^2^The implementation was carried out in Stata using the mi impute and mi estimate commands.

**Table E1:** Summary statistics, original datasets (EHIS and HBS)

| **Variable** | Obs | Mean | Std. dev. | Min | Max |
| --- | --- | --- | --- | --- | --- |
| **HBS** |  |  |  |  |  |
| Health expenditures | 12,419 | 117.22 | 255.90 | 0 | 5,711 |
| Gender (1=male) | 12,419 | 1.466 | 0.499 | 1 | 2 |
| Age group | 12,419 | 9.004 | 2.210 | 4 | 12 |
| Geographic location | 12,419 | 2.621 | 1.310 | 1 | 5 |
| Employment status | 11,607 | 1.636 | 0.607 | 1 | 3 |
| Education | 12,419 | 3.831 | 0.827 | 1 | 5 |
| Marital status | 12,419 | 2.081 | 1.393 | 1 | 4 |
| Income quintile | 12,419 | 3.256 | 1.392 | 1 | 5 |
| **EHIS** |  |  |  |  |  |
| Weight (kg) | 1,465 | 71.874 | 13.947 | 40 | 127 |
| Height (cm) | 1,467 | 170.995 | 9.136 | 140 | 195 |
| Gender (1=male) | 1,474 | 1.410 | 0.492 | 1 | 2 |
| Age group | 1,474 | 8.558 | 2.084 | 4 | 12 |
| Geographic location | 1,474 | 2.501 | 1.26 | 1 | 5 |
| Employment status | 1,474 | 1.754 | 0.693 | 1 | 3 |
| Education | 1,474 | 3.936 | 0.767 | 1 | 5 |
| Marital status | 1,474 | 1.847 | 1.232 | 1 | 4 |
| Income quintile | 1,474 | 3.769 | 1.151 | 1 | 5 |

**Notes:** Education (1= no formal education, 2= primary school, 3= lower middle school, 4= high school diploma, 5= undergraduate or postgraduate degree); Marital status (1= single, 2= married, 3= legally separated or di- vorced, 4 = widowed); Age (4= between 18 and 24 years, 5= between 25

and 29 years, 6= between 30 and 34 years, 7 = between 35 and 39 years,

8= between 40 and 44 years, 9=between 45 and 49 years, 10 = between 50

and 54 years, 11 = between 55 and 59 years, 12= between 60 and 64 years); Geographic location (1=northeast, 2= northwest, 3=centre , 4= south , 5= islands); Employment status (1=Executive, 2=Worker (subordinate), 3=self-employed).

**Table E2:** Summary statistics, matched dataset

| **Variable** | Obs | | Mean | Std. | dev. | Min | Max |
| --- | --- | --- | --- | --- | --- | --- | --- |
| Gender (1=male) |  | 13,893 | 1.46 | 0.50 | | 1 | 2 |
| Age group |  | 13,893 | 8.96 | 2.20 | | 4 | 12 |
| Geographic location |  | 13,893 | 2.61 | 1.31 | | 1 | 5 |
| Employment status |  | 13,081 | 1.65 | 0.62 | | 1 | 3 |
| Education |  | 13,893 | 3.84 | 0.82 | | 1 | 5 |
| Marital status |  | 13,893 | 2.06 | 1.38 | | 1 | 4 |
| Income |  | 13,893 | 3.31 | 1.38 | | 1 | 5 |
| Imputed height: sample mean |  | 9,634 | 170 | 8 | | 140 | 195 |
| Imputed health expenditures: | sample mean | 13,593 | 119.01 | 248.00 | | 0.00 | 5711 |
| Imputed health expenditures: | 1st quintile | 1,968 | 31.56 | 64.44 | | 0.00 | 709 |
| Imputed health expenditures: | 5th quintile | 3,431 | 241.68 | 408.23 | | 0.00 | 5711 |
| Imputed weight: sample mean |  | 8,379 | 71.58 | 12.42 | | 40 | 127 |
| Imputed weight: 1st quintile |  | 780 | 70.65 | 13.37 | | 40 | 120 |
| Imputed weight: 5th quintile |  | 2,604 | 72.59 | 12.63 | | 40 | 127 |

**Notes:** Education (1= no formal education, 2= primary school, 3= lower middle school, 4= high school diploma, 5= undergraduate or postgraduate degree); Marital status (1= single, 2= married, 3= legally separated or divorced, 4 = widowed); Age (4= between 18 and 24 years,

5= between 25 and 29 years, 6= between 30 and 34 years, 7 = between 35 and 39 years, 8=

between 40 and 44 years, 9=between 45 and 49 years, 10 = between 50 and 54 years, 11 =

between 55 and 59 years, 12= between 60 and 64 years); Geographic location (1=northeast, 2= northwest, 3=centre , 4= south , 5= islands); Employment status (1=Executive, 2=Worker (subordinate), 3=self-employed).

# Ad valorem tax

In addition to the main counterfactual experiment, we simulate a practical increase in the existing Value Added Tax (VAT) on fat-rich food groups such as cheese, processed meat, and sweets/snacks. These food groups are among the highest in saturated fat content. The proposed increase is designed to reduce fat consumption by 30%, which corresponds to a 4.3% rise in their initial prices. This adjustment is equivalent to introducing an ad valorem

(av) fat tax (t), where the after-tax price of a taxed food group j, denoted as *p^j^* , is:

1*,av*

*j*

*p*

1*,av*

= *p^j^* (1 + *tη^j^*) (4)

Here, *η^j^* represents the per-unit saturated fat content of food group j.

0

Since fat and cheese, processed meat and sweets and snacks differ both in the per kg content of saturated fat and in the compensated price elasticity of quantity, we compute the ad valorem tax that brings about a 30% decrease in saturated fat consumption as:

−0*.*30

*tη*¯ = *η*¯

*ϵ*¯

(5)

where *ϵ*¯ is the average of the own-price compensated elasticities of the three taxed food groups, and *η*¯ is the average of the saturated fat content per kg of fat and cheese, processed meat, sweets and snacks.

The top panel in Figure [F1](#_bookmark8) illustrates the distribution of the compensating variation resulting from the ad valorem tax, presented in both absolute monetary terms (a) and as a share of total expenditure (b). Panels (c) and (d) in Figure [F1](#_bookmark8) depict the distribution of benefits in €/month, and relative to total expenditure, respectively. Finally, panel (e) in Figure [F1](#_bookmark8) displays the net impact on consumer welfare.

**Figure F1:** Costs and Benefits, ad valorem tax

**(a)** CV (€/month) **(b)** CV/total expenditure


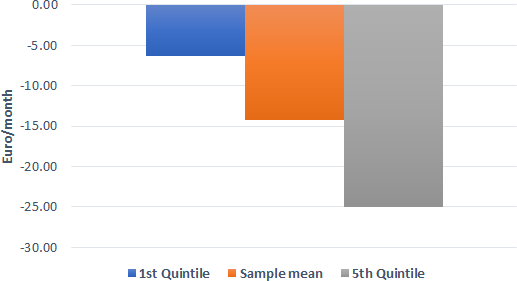

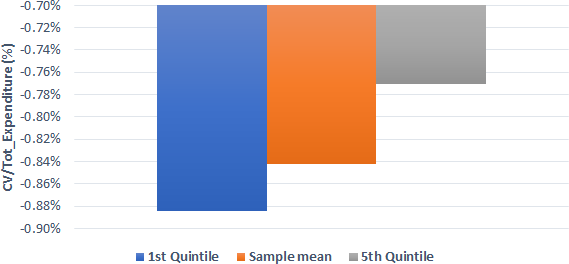


**(c)** Health benefits (€/month) **(d)** Health benefits/total expenditure


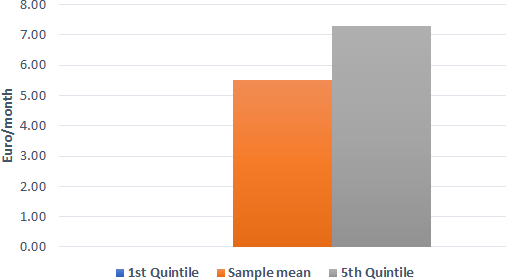

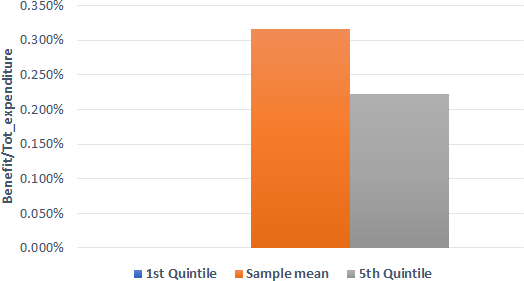


**(e)** Net welfare effects, ad valorem tax


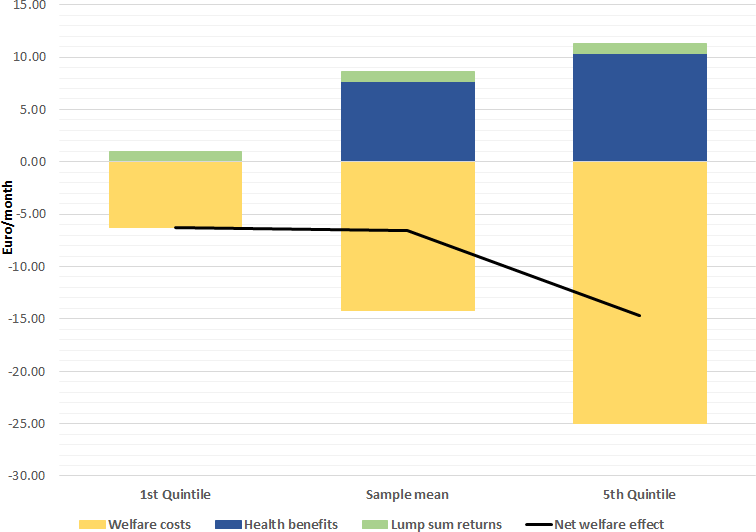


**Notes**: Figures a-b show welfare costs in €/month (a) and as a fraction of total expenditure (b). Figures c-d show health benefits in €/month (c) and as a fraction of total expenditure (d). Figure e decomposes welfare changes resulting from the ad valorem tax across the distribution of total expenditure. “Welfare costs” are measured by the compensating variatio2n1 (€/month). ”Health benefits” are calculated as

savings in health expenditures (€/month) due to weight loss. ”Lump sum return” is public revenues

(€/month) from the fat tax redistributed equally across the distribution of total expenditure. The bold black line is the ”Net welfare effect”, i.e. the difference between ”Welfare costs” and ”Health benefits”.

# References

Alpman, A. (2016). Implementing rubin’s alternative multiple-imputation method for statistical matching in stata. *The Stata Journal 16* (3), 717–739.

Irz, X. (2017). Demand for food and nutrients and its climate impact: A micro-econometric analysis of economic and socio-demographic drivers. Natural Resources and Bioeconomy Studies 28, Natural Resources Institute Finland, Helsinki.

Rubin, D. B. (1986). Statistical matching using file concatenation with adjusted weights and multiple imputations. *Journal of Business & Economic Statistics 4* (1), 87–94.

Wooldridge, J. W. (2010). *Econometric Analysis of Cross Section and Panel Data (Second Edition)*. The MIT Press.

22

1. When estimated at the sample mean, Marshallian elasticities are computed as

$$\frac{\partial{lnq}^{i}}{\partial{lnp}^{j}}=\frac{\alpha^{ij}}{\bar{w}^{i}}-\delta_{ij}-\frac{\bar{w}^{j}}{\bar{w}^{i}}\left[ \sum_{r=1}^{R} b_{r}^{i}r\left( \hat{\bar{y}} \right)^{r-1} \right]$$

 [↑](#footnote-ref-1)
